# Cryptic Diversity and Genetic Differentiation of Mesophotic Hydroids in the Southwestern Indian Ocean

**DOI:** 10.1002/ece3.72665

**Published:** 2025-12-17

**Authors:** David Ory, Nicole Gravier‐Bonnet, Pascale Chabanet, Chloé A. F. Bourmaud, Emilie Boissin

**Affiliations:** ^1^ UMR9220 ENTROPIE – Université de La Réunion, IRD, CNRS, IFREMER Université de Nouvelle‐Calédonie Saint‐Denis La Réunion France; ^2^ PSL Research University, CNRS‐EPHE‐UPVD, UAR3278 CRIOBE Perpignan France; ^3^ Association Poisson Lune Le Tampon La Réunion France; ^4^ Association Vie Océane Saint‐Paul La Réunion France; ^5^ Laboratoire d'Excellence CORAIL, Papetoai Moorea French Polynesia

**Keywords:** connectivity, Mayotte, microsatellite, Reunion, species complex, twilight zone

## Abstract

The western Indian Ocean (WIO) is recognized as a marine biodiversity hotspot with complex oceanographic circulation resulting in limited connectivity between remote islands. This ocean region comprises several subregions of varying biodiversity, with the northern Mozambique Channel standing out as the core of this hotspot. Although the hydroids in this region are known to include cryptic species and show contrasting connectivity patterns, the mesophotic depths remain largely unexplored. The Deep Reef Refuge Hypothesis suggests that mesophotic coral ecosystems may act as refuges. However, this hypothesis is based on several prerequisites that could be affected by the presence of cryptic species. We investigated the genetic diversity and connectivity of seven hydroid species by collecting samples at euphotic and mesophotic depths around the islands of Mayotte and Reunion. Population genetic patterns were investigated using multivariate analyses and Bayesian clustering, with 8–18 microsatellite markers per species. The results revealed greater genetic diversity in Mayotte than in Reunion, even though fewer samples were collected there. This is in line with the location of the heart of the hotspot in the northern part of the WIO. In addition, all species exhibited strong genetic differentiation between samples from the two islands, supporting the “one island, one species” hypothesis previously proposed for hydroids in the region. However, contrasting values were obtained among depths depending on the species and the island, demonstrating the importance of a multi‐species approach. The inclusion of mesophotic samples from the *Taxella eximia/gracilicaulis* and *Macrorhynchia phoenicea* species complexes provides new insights into the true biodiversity of these genera, revealing additional cryptic species and putative hybridization. Furthermore, the genetic connectivity estimation performed here among depths highlights several species that could be evaluated in terms of the vertical connectivity prerequisite of the Deep Reef Refuge Hypothesis in Mayotte and Reunion.

## Graphical abstract

To assess whether hydroids (Cnidaria, Hydrozoa) are good candidates to test the Deep Reef Refuge Hypothesis and particularly the vertical connectivity component, we analysed the genetic diversity and structure of seven species sampled across euphotic and mesophotic depths around Mayotte and Reunion in the southwestern Indian Ocean. Consistent with the position of the western Indian Ocean's biodiversity hotspot, genetic diversity was higher in Mayotte than in Reunion, while all species showed strong genetic differentiation between islands. Incorporating mesophotic samples not only uncovered additional cryptic lineages, but also revealed depth‐related genetic patterns that varied sharply among species and between islands.

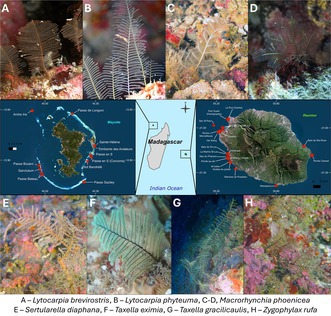

## 1 Introduction

Coral reefs provide important ecosystem services worldwide (Woodhead et al. 2019; Eddy et al. 2021), yet they are globally threatened (Baker et al. 2008; Hughes et al. 2017; Hughes, Anderson, et al. 2018; Hughes, Kerry, et al. 2018). Although the Indian Ocean remains one of the least studied ocean basins, coral reefs are vital to many tropical countries, including those in the Western Indian Ocean (Wafar et al. 2011; Obura et al. 2022). This region is recognized as a biodiversity hotspot (Roberts et al. 2002; Wafar et al. 2011; Marchese 2015), supported by studies of corals, reef fishes, reef brittle stars, and giant clams (Obura 2012; Hoareau et al. 2013; McClanahan et al. 2014; Borsa et al. 2016; Fauvelot et al. 2020). Coral reefs in the Western Indian Ocean are now severely threatened (Obura et al. 2022), and given their importance to the sustainability of nearby human populations, urgent and continued efforts are needed to better understand and safeguard their global functioning.

Population genetics is a powerful tool for conservation in marine environments that lack clear boundaries (O'Brien 1994; Avise 1998; Ouborg 2010; Mertens et al. 2018). Marine connectivity is driven by currents, and the Southwestern Indian Ocean (SWIO), traditionally defined between the Seychelles in the north, the Mascarene Islands in the east, the south coast of Madagascar in the south, and the East African coastal countries (South Africa, Mozambique, Tanzania, Kenya) in the west (fig. 1 from Obura et al. 2022), is characterized by complex oceanographic processes (Schott et al. 2009; Vogt‐Vincent and Johnson 2023). The SWIO is divided into several subregions, with low connectivity between them on short time scales (Crochelet et al. 2016; Gamoyo et al. 2019; Vogt‐Vincent et al. 2024). In this context, population genetic studies highlight the high genetic diversity and contrasting connectivity in this region according to several models: corals (Vogler et al. 2012; van der Ven et al. 2021, 2022; Burt et al. 2024), sea stars (Otwoma and Kochzius 2016), and reef brittle stars (Hoareau et al. 2013; Boissin et al. 2017). While hydroids (Cnidaria, Hydrozoa) have usually been neglected in studies of coral reefs compared with corals (Di Camillo et al. 2017), several studies focused on them in the SWIO. Population genetic studies of SWIO hydroids have identified several cryptic species in different subregions, supporting the hidden diversity and low connectivity observed in this region (Postaire et al. 2016; Postaire, Gélin, Bruggemann, and Magalon 2017; Postaire, Gélin, Bruggemann, Pratlong, and Magalon 2017; Boissin et al. 2018). The observation of cryptic species tied to different islands has inspired the hypothesis that there could be one species per island: the “one island, one species” hypothesis (Postaire, Gélin, Bruggemann, and Magalon 2017; Boissin et al. 2018).

Recently, mesophotic coral ecosystems, located between 30 and 150 m depth (Loya et al. 2019), have been investigated for the Deep Reef Refuge Hypothesis (DRRH), that is, their potential to act as refuges for the shallower threatened reefs (Bongaerts et al. 2010). However, it needs to be further validated by testing two main prerequisites: (1) the presence of the same species at euphotic and mesophotic depths and (2) vertical connectivity among their populations. However, most mesophotic studies do not account for possible speciation processes at mesophotic depths (Glazier and Etter 2014; Prada and Hellberg 2021). Cryptic species may be present at these depths, which does not strictly validate the first prerequisite and subsequently may affect the estimation of vertical connectivity (second prerequisite), which must be taken into account when testing the DRRH. Evaluations of the second prerequisite of the DRRH indicate that it is not universally supported and appears to be both taxon‐ and region‐dependent (Bongaerts et al. 2017; Morais and Santos 2018; Medeiros et al. 2021; Loiseau et al. 2023). Moreover, this prerequisite requires further testing, particularly in light of the potential presence of cryptic species.

Mesophotic depths in the SWIO remain largely understudied (Turner et al. 2017; Eyal et al. 2021). Existing studies have primarily focused either on documenting the mesophotic distribution of known species or on discovering new taxa at these depths (Tea et al. 2019; Boissin et al. 2021; Hoarau et al. 2021; Osuka et al. 2021; Muff et al. 2023). In contrast, comparisons between euphotic and mesophotic depths to assess their potential to act as refuges are rare and not based on genetic methods (Loiseau et al. 2023; Stefanoudis et al. 2023). Recent explorations of mesophotic coral ecosystems around Reunion highlighted the unknown diversity of hydroids (Gravier‐Bonnet et al. 2022). Notably, several hydroid species occur at both euphotic and mesophotic depths, making them promising candidates for testing the vertical connectivity prerequisite of the DRRH. Furthermore, genetic markers are already available for some of these species, providing a robust framework for assessing depth‐related population connectivity (Postaire et al. 2015a, 2015b; Ory, Gravier‐Bonnet, et al. 2025; Ory, Mouronvalle, et al. 2025).

This study investigates the genetic diversity and both horizontal and vertical connectivity of seven widespread hydroid species in the SWIO. Specimens were collected from both euphotic and mesophotic depths at two geographically distant locations, Reunion and Mayotte, allowing for the assessment of regional (inter‐island) and depth‐related (intra‐island) genetic structuring.

## 2 Materials and Methods

### 2.1 Species and Sampling Sites

**FIGURE 1:**
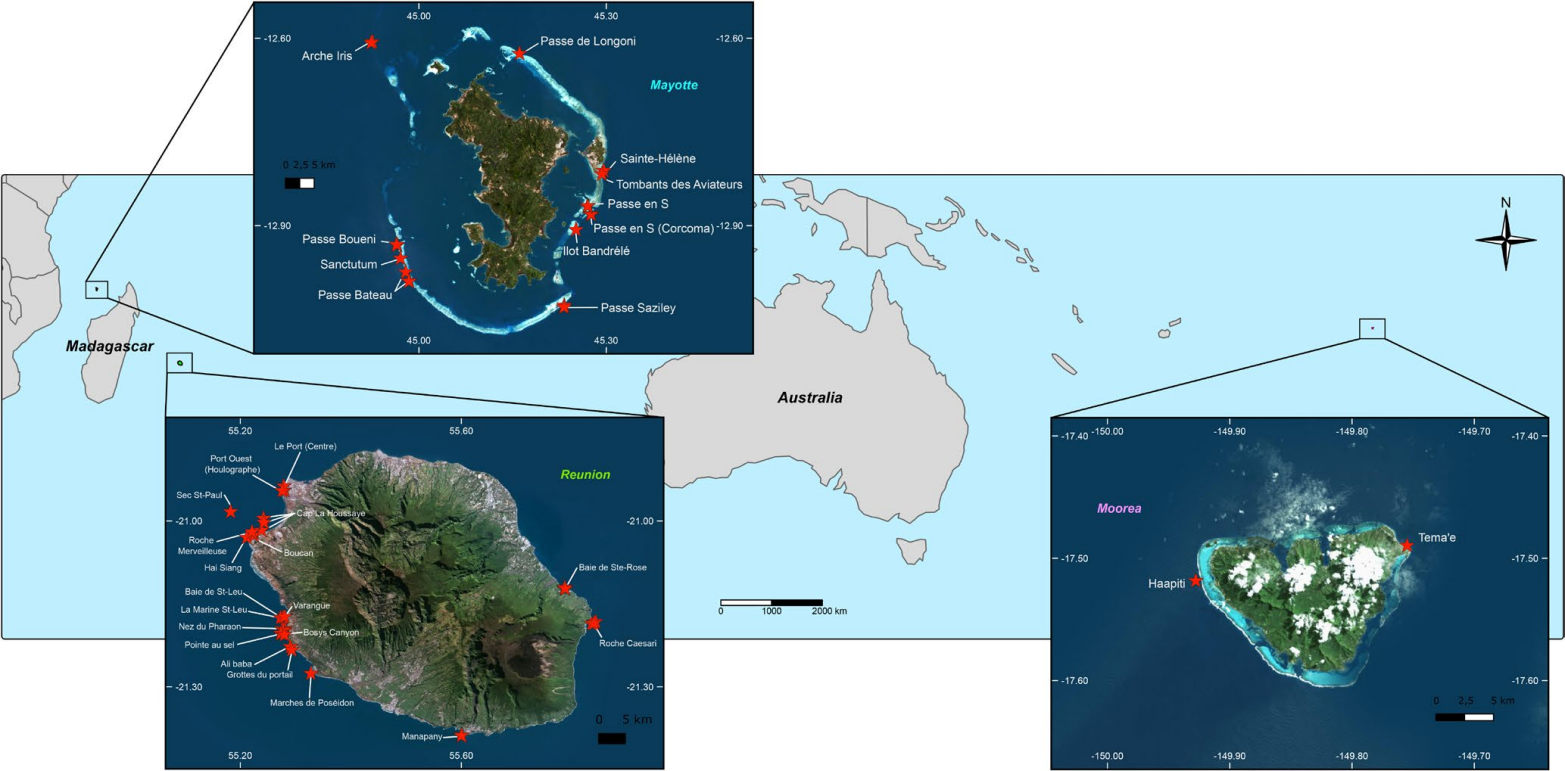
Map of Reunion and Mayotte in the Indian Ocean, and Moorea in the Pacific Ocean. A total of 1570 samples were collected from 35 sites on Reunion, 19 on Mayotte, and 2 on Moorea at depths ranging from 13 to 104 m.

Seven hydroid species were selected for this study: *Lytocarpia brevirostris* (Busk, 1852), *Lytocarpia phyteuma* (Stechow, 1919), *Macrorhynchia phoenicea* (Busk, 1852), *Taxella gracilicaulis* (Jäderholm, 1903), *Taxella eximia* Allman, 1874 (Aglaopheniidae), *Sertularella diaphana* (Allman, 1885) (Sertularellidae), and *Zygophylax rufa* (Bale, 1884) (Zygophylacidae). Sampling was carried out between 2020 and 2023 around two islands in the SWIO: Mayotte (Comoros Archipelago) and Reunion (Mascarene Archipelago), and the island of Moorea in French Polynesia in the Pacific Ocean (for *L. phyteuma*). A total of 35 sites were sampled on Reunion, 19 on Mayotte, and 2 on Moorea at depths ranging from 13 to 104 m (Figure 1). Sampling at mesophotic depths (below 40 m) was performed using closed‐circuit rebreathers (CCR) with gas mixes adapted to the targeted depths (hypoxic trimix diving techniques). Previous mesophotic studies highlight a break between upper and lower mesophotic depths (Lesser et al. 2019; Laverick et al. 2020), and given the light intensity at these depths around Reunion (Hoarau et al. 2024), individuals were classified according to their sampling depth: above 40 m = euphotic (Eu), between 40 and 70 m = upper mesophotic (UM), and between 70 and 104 m = lower mesophotic (LM).

A total of 1570 samples were collected, 1215 from Reunion, 307 from Mayotte, and 48 from Moorea. A colony fragment was preserved in 96% ethanol for molecular analysis, and a subset of representative specimens (whole colonies with species‐typical morphology and reproductive structures, when available) was preserved in a 3.6% saline formaldehyde solution for taxonomic studies. The genotypes from the 1215 Reunion samples of *L. brevirostris* , *L. phyteuma*, *M. phoenicea*, *S. diaphana* , and *Z. rufa* were obtained from a study focusing on vertical connectivity around Reunion (Ory, Gravier‐Bonnet, et al. 2025). They were analyzed simultaneously with the samples from Mayotte and Moorea, as described below.

Two cryptic species of *M. phoenicea*, *⍺* and *ꞵ*, were identified in the SWIO according to Postaire et al. (2016). These cryptic species were distinguished by shape and color: stiff and black and white (cryptic species *⍺*), and gracile and brown (cryptic species *β*), and were genetically distinct (*F*_ST_ = 0.33 from Postaire et al. 2016).

### 2.2 DNA Extraction and Microsatellite Amplification

DNA was extracted in columns using the DNeasy Blood and Tissue Kit or the QIAamp 96 DNA Kit for the QIAcube HT robot extractor (Qiagen, Hilden, Germany) according to the manufacturer's protocols. Samples were genotyped using microsatellite markers developed specifically for the species studied here (Postaire et al. 2015a, 2015b; Ory, Mouronvalle, et al. 2025). A total of 83 markers were amplified using the same multiplex panels and annealing temperature as those used for their development (Postaire et al. 2015a, 2015b; Ory, Mouronvalle, et al. 2025), with the PCR cycling parameters used in Ory, Mouronvalle, et al. (2025). Briefly, amplifications were performed using the Type‐it Microsatellite PCR Kit (Qiagen, Hilden, Germany) in an 11‐μL total volume: 5‐μL Type‐it Multiplex PCR Master Mix [2X], 1‐μL Q solution, 0.01 μL of each primer [100 μM], 3.98‐μL RNase‐free water and 1‐μL DNA extract. PCR program is settled as follows: an initial denaturation step of 5 min at 95°C, followed by 40 cycles consisting of: (i) 30 s at 95°C, (ii) 1 min 30 s at optimal annealing temperature for each locus (53°C–60°C, see tab. 1 from Ory, Mouronvalle, et al. 2025), (iii) 30 s at 72°C, and a final extension step at 60°C for 30 min. PCR products were visualized on 2% agarose gels and sent to GenoScreen (Lille, France) for genotyping. Allele sizes were manually scored using Geneious Prime software v.2022.2.2 (Biomatters, San Diego, USA) and ambiguous peaks were considered as missing data. Individuals with readable peaks were considered successfully genotyped, and the alleles were kept in the final analyzed dataset.

### 2.3 Verification of Genotypes

The presence of clones among genotypes was checked using the R software v.4.3 (R Core Team 2023) with the RStudio interface v.2024.04 (Posit Team 2024) and the R packages *poppr* v.2.9.6 (Kamvar et al. 2014) and *adegenet* v.2.1.10 (Jombart 2008) with the “*isPoly*” and “*mlg*” functions. After removing duplicate genotypes within each island, unique genotypes (MLG) were retained in the following analyses (Data S1).

### 2.4 Data Analysis

Mean number of alleles per locus (*M*_NA_), observed (*H*_o_) and expected (*H*_e_) heterozygosities, and fixation indices (*F*_IS_) were calculated using the R packages *poppr* and *hierfstat* v.0.5‐11 (Goudet and Jombart 2022). The significance of global fixation indices (*F*_IS_) by islands per species was estimated using a bootstrap method implemented in the *hierfstat* package with 100,000 repetitions. To account for the unbalanced number of samples between islands, the mean number of alleles per locus was also calculated using a rarefaction approach estimated by the “*allelic richness*” function from the *hierfstat* package. The rarefied mean number of alleles (R‐*M*_NA_) was estimated from the break point in the rarefaction curves (*M*_NA_~number of alleles sampled, data not shown). The number of private alleles per island (*N*_PA_) was estimated using GenAlEx v.6.503 (Peakall and Smouse 2012). Again, to account for the unbalanced number of samples between islands, a random sampling of individuals was performed to obtain populations of equal size, with the number of samples corresponding to the lowest population size available (i.e., the lowest MLG per island for each species, Table 1). Similar random sampling was performed 100 times per species, and for each repetition, the number of private alleles per island was estimated for the sampled populations using the “*private_alleles*” function from the *poppr* package. The rarefied value of private alleles per island (R‐*N*_PA_) was calculated by averaging the number of private alleles estimated in the 100 repetitions.

**TABLE 1:** Summary of diversity metrics per island for the seven hydroid species.

	NL	Island	MLG	*M*_NA_ (R‐*M*_NA_)	*N*_A_	*N*_PA_ (R‐*N*_PA_)	*H*_o_	*H*_e_	*F*_IS_
*Lytocarpia brevirostris*	8	Reunion	166	5.75 (4.92)	46	11 (9)	0.367	0.398	0.055*
		Mayotte	82	10.25 (10.17)	82	47 (51)	0.476	0.588	0.188*
*Lytocarpia phyteuma*	16 (13)	Reunion	41	4.13 (3.30)	62	23 (23)	0.442	0.475	0.084
		Mayotte	37	6.19 (5.11)	99	53 (54)	0.562	0.717	0.262
		Moorea	48	6.23 (4.01)	81	56 (52)	0.396	0.454	0.113
*Macrorhynchia phoenicea*	15	Reunion	141	8.60 (5.44)	129	91 (50)	0.375	0.591	0.390*
		Mayotte	21	4.40 (4.40)	66	28 (37)	0.127	0.466	0.763*
*Sertularella diaphana*	18	Reunion	121	8.94 (5.28)	161	79 (36)	0.501	0.572	0.153*
		Mayotte	15	6.72 (6.36)	121	39 (59)	0.525	0.692	0.268*
*Taxella gracilicaulis*	14	Reunion	67	7.86 (3.61)	110	71 (66)	0.230	0.541	0.317*
		Mayotte	53	7.64 (3.99)	107	68 (69)	0.387	0.628	0.658*
*Taxella eximia*	14	Reunion	42	4.50 (2.46)	63	30 (27)	0.167	0.487	0.690*
		Mayotte	32	6.43 (3.24)	90	57 (60)	0.296	0.663	0.578*
*Zygophylax rufa*	12	Reunion	369	19.42 (4.38)	233	167 (69)	0.361	0.621	0.444*
		Mayotte	23	8.42 (4.92)	101	35 (69)	0.465	0.743	0.383*

*Note:* Metrics are highlighted for Reunion in green, Mayotte in blue, and Moorea in purple.

Abbreviations: *F*_IS_, coefficient of inbreeding; *H*_e_, Expected heterozygosity; *H*_o_, observed heterozygosity; MLG, number of unique genotypes; *M*_NA_, mean number of alleles per locus; NL, number of microsatellite loci; *N*_PA_, number of private alleles; R‐*M*_NA_, rarefied mean number of alleles per locus; R‐*N*_PA_, rarefied number of private alleles.

* Indicates significant *p*‐values (< 0.05).

The genetic differentiation values (*F*_ST_) between islands were estimated using GenAlEx. The significance of the pairwise *F*_ST_ between islands for each species was estimated using 999 permutations in GenAlEx. For *L. phyteuma*, only 13 loci were used for comparisons between the three islands (Lp21, Lp26, Lp37 did not amplify in samples from Moorea). For three species: *L. brevirostris* , *L. phyteuma*, and *T. gracilicaulis*, pairwise *F*_ST_ between depth classes (euphotic = Eu, upper mesophotic = UM, lower mesophotic = LM) were estimated using GenAlEx (999 permutations), and for each comparison, *p*‐values were manually adjusted using the Benjamini and Hochberg false discovery rate correction (BH, Benjamini and Hochberg 1995). To estimate *F*_ST_ between depth classes, some loci had to be removed from different datasets when they were not amplified in the samples of certain islands. For *L. phyteuma*, one locus was removed for each dataset: Lp37 for the Reunion dataset and Lp23 for the Mayotte dataset. Additionally, on Mayotte, only one sample was collected from LM depths, and it was decided to combine it with the UM samples. For *T. gracilicaulis*, two loci were removed for each dataset: Tg14, Tg29 for the Reunion dataset and Tg05, Tg29 for the Mayotte dataset.

To visualize the differentiation between islands, principal coordinate analysis (PCoA) was performed with the R packages *ade4* v.1.7‐22 (Thioulouse et al. 2018) and *adegenet* using Nei's genetic distance. The Structure software v.2.3.4 (Pritchard et al. 2000), Evanno's Δ*K*, calculated by the Structure Harvester program (Evanno et al. 2005; Earl and vonHoldt 2012), and the log likelihood Ln Pr(X|*K*) estimated by Structure (Pritchard et al. 2000; Janes et al. 2017), were used to explore population structure and determine the most likely number of genetic clusters. For each species, analyses were performed with no prior and with the sampling island and/or depth as prior. Simulations were run with an assumed number of populations (*K*) from 1 to 10, 10 replicates for each value of *K*, and 1,000,000 Markov Chain Monte Carlo (MCMC) with a burn‐in length of 100,000 chains per simulation.

Regarding the sampling of *M. phoenicea* cryptic species performed in the present study, we decided to perform analyses with a dataset combining the two cryptic species, with additional estimation of *F*_ST_ between cryptic species and Structure analyses with cryptic species as prior. For this species only, separate analyses of molecular variance (AMOVA) were performed using the R packages *poppr* and *ade4* to determine the main factor explaining genetic differentiation between islands, depths, or cryptic species.

For *Taxella* species, individuals were separated by species only for the PCoA and Structure simulations: *T. gracilicaulis*, *T. eximia* , and a third group, *T. eximia/gracilicaulis*, with 13 specimens that have the morphological characteristics of *T. eximia* (Ronowicz et al. 2017) but the global shape of *T. gracilicaulis* (large colony with distant ramifications) as well as by island and depth. For the remaining analyses, these 13 specimens were considered to be *T. eximia* specimens, including the additional estimation of pairwise *F*_ST_ among the species.

## 3 Results

### 3.1 Genetic Diversity

Of the 1570 hydroid samples successfully genotyped, a total of 1258 MLG were retained for analyses (947 from Reunion, 263 from Mayotte, 48 from Moorea) (Table 1). For the seven target species, the rarefied mean number of alleles (R‐*M*_NA_) ranged from 2.46 to 5.44 for Reunion and 3.24 to 10.17 for Mayotte. The rarefied number of private alleles (R‐*N*_PA_) was estimated to range from 9 to 69 for Reunion. The rarefied number of private alleles was globally higher in Mayotte than in Reunion, with R‐*N*_PA_ ranging from 37 to 69 (Table 1).

The observed heterozygosities (*H*_o_) ranged from 0.167 to 0.501 for Reunion and 0.127 to 0.562 for Mayotte (Table 1). The expected heterozygosities (*H*_e_) were higher than the observed heterozygosities, ranging from 0.398 to 0.621 and 0.466 to 0.743 for Reunion and Mayotte, respectively (Table 1). The fixation indices were significant for six species on Reunion and Mayotte: *Lytocarpia brevirostris* (*F*_IS_ = 0.05 and 0.19 for Reunion and Mayotte, respectively), *Macrorhynchia phoenicea* (*F*_IS_ = 0.39 and 0.76), *Sertularella diaphana* (*F*_IS_ = 0.15 and 0.27), *Taxella gracilicaulis* (*F*_IS_ = 0.32 and 0.66), *Taxella eximia* (*F*_IS_ = 0.69 and 0.58) and *Zygophylax rufa* (*F*_IS_ = 0.44 and 0.38) (Table 1). The *F*_IS_ values for these species were higher in Mayotte than in Reunion (except for *T. eximia* and *Z. rufa* ). These values suggest the existence of population structures on the two islands, which will be estimated in the following analyses. For *Lytocarpia phyteuma*, the fixation indices were not significant for any of the three islands. The rarefied mean number of alleles (R‐*M*_NA_ = 4.01), the rarefied number of private alleles (R‐*N*_PA_ = 52), and the observed (*H*_o_ = 0.40) and expected (*H*_e_ = 0.45) heterozygosities for *L. phyteuma* samples from Moorea were in the same range as those for Reunion and Mayotte (Table 1).

### 3.2 Population Structure Within Species

For the species that do not include cryptic species: *L. brevirostris* , *L*. *phyteuma*, *S. diaphana* , and *Z. rufa* , genetic differentiation values (*F*_ST_) were significant between populations of Reunion (R) and Mayotte (M) (*F*_ST__R‐M = 0.14–0.22), and for *L. phyteuma* between populations of Moorea (Mo) and Reunion (*F*_ST__R‐Mo = 0.32) or Mayotte (*F*_ST__M‐Mo = 0.28) (Table 2). Genetic differentiation values between depths were contrasted between species and islands. For *L. brevirostris* , *F*_ST_ between depths was not significant, except between euphotic and upper mesophotic depths (*F*_ST__Eu‐UM = 0.008), while they were higher in Mayotte. However, they were still weak (*F*_ST_ = 0.025–0.032), and equivalents between the different depths (Eu vs. UM vs. LM) (Table 3). For *L. phyteuma*, the *F*_ST_ between euphotic and mesophotic depths (upper and lower mesophotic depths combined) was higher in Mayotte (*F*_ST_ = 0.137) than in Reunion (*F*_ST_ = 0.079) (Table 3). However, as no mesophotic samples of *L. phyteuma* have been collected in Moorea, it was not possible to estimate F_ST_ between euphotic and mesophotic depths (Table 3). For *S. diaphana* and *Z. rufa* , the *F*_ST_ values between depths could not be estimated in Mayotte due to the small number of samples collected. In Reunion, the *F*_ST_ between depths for *S. diaphana* was not significant or weak between euphotic and lower mesophotic depths (*F*_ST__Eu‐LM = 0.038) (Table 3), whereas for *Z. rufa* , *F*_ST_ between adjacent depths was lower than between distant depths (Table 3). Results for Reunion are the subject of a specific paper focusing on vertical connectivity using the same individuals (Ory, Gravier‐Bonnet, et al. 2025).

**TABLE 2:** Pairwise genetic differentiation values (*F*_ST_) between islands.

Species		Reunion (R)		Mayotte (M)
		Mayotte (M)	Moorea (Mo)	
	NL	*F*_ST__R‐M	*F*_ST__R‐Mo	*F*_ST__M‐Mo
*Lytocarpia brevirostris*	8	0.220*	—	—
*Lytocarpia phyteuma*	13	0.197*	0.320*	0.227*
*Macrorhynchia phoenicea*	15	0.203*	—	—
*Sertularella diaphana*	18	0.140*	—	—
*Taxella gracilicaulis*	14	0.208*	—	—
*Taxella eximia*	14	0.212*	—	—
*Zygophylax rufa*	12	0.160*	—	—

Abbreviations: *F*_ST__M‐Mo, between Mayotte and Moorea; *F*_ST__R‐M, between Reunion and Mayotte; *F*_ST__R‐Mo, between Reunion and Moorea; NL, number of microsatellite loci.

* Indicates significant *p*‐values (< 0.05).

**TABLE 3:** Pairwise genetic differentiation values (*F*_ST_) between depth classes.

	NL	Island	*F*_ST__Eu‐UM	*F*_ST__Eu‐LM	*F*_ST__UM‐LM
*Lytocarpia brevirostris*	8	Reunion	0.008*	0.019	0.016
		Mayotte	0.025*	0.026*	0.032*
*Lytocarpia phyteuma*	15	Reunion	0.079*	—	—
		Mayotte	0.137*		—
*Taxella gracilicaulis*	12	Reunion	0.037*	0.070*	0.039*
		Mayotte	0.165*	0.233*	0.060*
*Sertularella diaphana*	18	Reunion	0.025	0.038*	0.121
*Zygophylax rufa*	12	Reunion	0.056*	0.178*	0.049*
*Macrorhynchia phoenicea*	15	Reunion	0.078*	0.132*	0.017*

*Note:* For *L. phyteuma*: Lp37 was removed for the Reunion dataset and Lp23 for the Mayotte dataset. Only one sample was collected from LM depths, it was combined with the UM samples. For *T. gracilicaulis*: Tg14, Tg29 were removed for the Reunion dataset and Tg05, Tg29 were removed for the Mayotte dataset. *F*_ST_ values are highlighted for Reunion in green, and for Mayotte in blue.

Abbreviations: *F*_ST__Eu‐LM, between euphotic and lower mesophotic depths; *F*_ST__Eu‐UM, between euphotic and upper mesophotic depths; *F*_ST__UM‐LM, between upper and lower mesophotic depths.

* Indicates significant *p*‐values (< 0.05).

**FIGURE 2:**
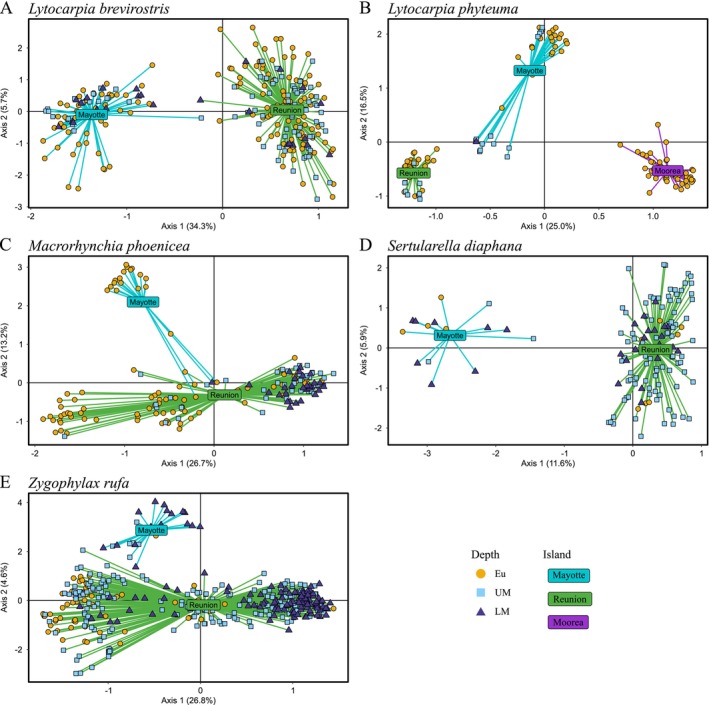
Principal coordinate analysis (PCoA) of five species. (A) *Lytocarpia brevirostris*, (B) *Lytocarpia phyteuma*, (C) *Macrorhynchia phoenicea* (*⍺* and *ꞵ*), (D) *Sertularella diaphana* , and (E) *Zygophylax rufa*. Individuals are colored by sampling depth: Eu = euphotic in yellow, UM = upper mesophotic in light blue, and LM = lower mesophotic in dark blue. Individuals are connected by colored lines, cyan for Mayotte, green for Reunion, purple for Moorea, to island centroids. Variance explained by the axis is given in brackets.

PCoAs clearly separated individuals of *L. brevirostris* , *L. phyteuma*, and *S. diaphana* by islands (Figure 2A,B,D). For *Z. rufa*, some individuals from Mayotte were close to individuals from Reunion (Figure 2E). However, considering the different depths, the PCoA of *L. brevirostris* and *S. diaphana* did not separate individuals by depth (Figure 2A,D), whereas for *L. phyteuma* the PCoA separated individuals between euphotic and mesophotic depths on the second axis, especially for those from Mayotte (Figure 2B).

**FIGURE 3:**
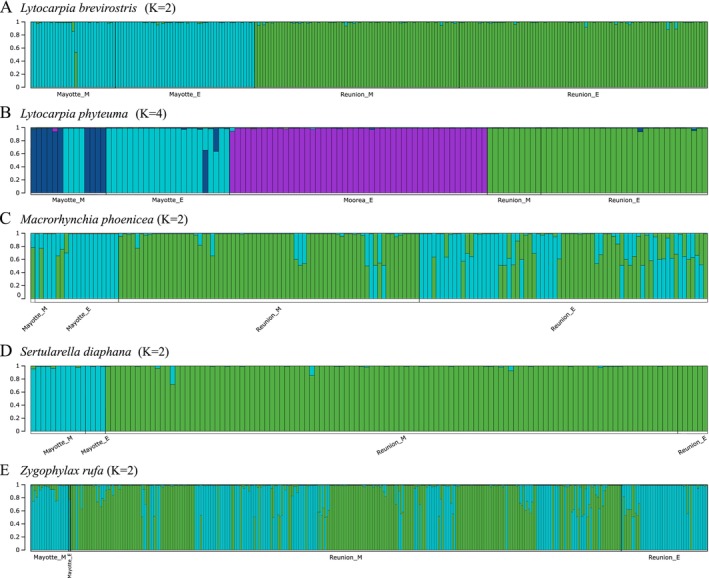
Assignment plots for five species without prior using *K* estimated by Evanno's Δ*K* and log likelihood Ln Pr(*X*|*K*) for each species (Table S1). (A) *Lytocarpia brevirostris*, (B) *Lytocarpia phyteuma*, (C) *Macrorhynchia phoenicea* (*⍺* and *ꞵ*), (D) *Sertularella diaphana* , and (E) *Zygophylax rufa*. Individuals are sorted by island and by depth, with the assumed population of Reunion colored in green, Mayotte in cyan, and Moorea in purple.

**FIGURE 4:**
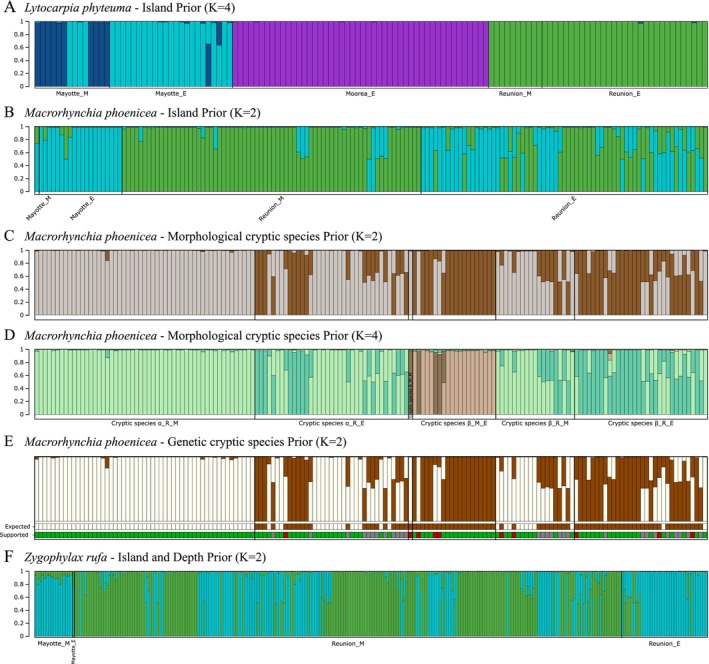
Assignment plots for three species with priors. Structure runs with islands as prior for *Lytocarpia phyteuma*, *Macrorhynchia phoenicea* (*⍺* and *ꞵ*), and *Zygophylax rufa*, and with cryptic species of *M. phoenicea* as prior using the *K* estimated by Evanno's Δ*K* and log likelihood Ln Pr(*X*|*K*) (Table S2). (A) For *L. phyteuma* with *K* = 4 with islands as prior. (B–E) For *M. phoenicea*: (B) *K* = 2 with islands as prior, (C) *K* = 2 and (D) *K* = 4 with morphological cryptic species as prior, (E) *K* = 2 with genetic cryptic species as prior: The “Expected” line was colored according to the expected PcoA attribution of species and the “Supported” line was colored if it was supported by structure assignment in green, not supported in red and ambiguous in gray. (F) For *Z. rufa* *K* = 2 with islands and depths as prior. Individuals are sorted by island and depth, with the assumed Reunion assignment colored in green, Mayotte in cyan and Moorea in purple. For the assignment plots of *M. phoenicea* with morphological cryptic species as prior, the individuals are sorted by cryptic species (with the *⍺* in gray and *ꞵ* in brown), by island and depth.

Estimates of the most likely number of populations determined by Evanno's Δ*K* and log likelihood Ln Pr(*X*|*K*) methods were similar, except for some species ( *S. diaphana* and *Z. rufa* ) for which the log likelihood method does not show a clear plateau (Figure S1). In these cases, the values retained are those of Evanno's Δ*K* (Tables S1 and S2). Thus, the most likely number of populations estimated was *K* = 2 for *L. brevirostris* , *S. diaphana* and *Z. rufa* , and *K* = 4 for *L. phyteuma* (Figure S1, Tables S1 and S2). For *L. brevirostris* and *S. diaphana*, these estimated clusters (*K* = 2) clearly correspond to the island populations (Figure 3A,D). However, for the two other species (*L. phyteuma* and especially *Z. rufa* ), the estimated cluster did not clearly correspond to the island population. For *L. phyteuma*, structure assignments separated individuals from Reunion, Moorea, and Mayotte. However, individuals from Mayotte were further separated by sampling depth, with a euphotic and a mesophotic cluster, and admixture between them with four mesophotic individuals assigned to the euphotic cluster (Figures 3B and 4A). For *Z. rufa* , the estimated clusters (*K* = 2) did not correspond to the island populations, either with or without islands as prior (Figures 3E and 4F). The individuals from Mayotte (both euphotic and mesophotic) were clustered together, as well as those from Reunion (Figures 3E and 4F). The Reunion individuals were separated into the two clusters that tend to assign individuals by depth, but with admixture between the clusters.

### 3.3 Genetic Differentiation and Structure of *Macrorhynchia phoenicea* Species Complex

In the present study, the two cryptic species of *M. phoenicea* are mainly identified by coloration (*⍺* = black and white, *β* = brown), which can be variable. Examination of the reproductive structures: size and shape of the phylactocarps (Gravier‐Bonnet N. personal observation) also helps to separate the two species, but this distinguishing character was not found in all our samples.

The cryptic species *⍺* was found only on Reunion, often at mesophotic depths, whereas species *β* was found only at euphotic depths on Reunion and at all depths on Mayotte. The genetic differentiation value between the two cryptic species, mixing Reunion and Mayotte samples, was significant but low (*F*_ST_ = 0.075, *p*‐value < 0.001) and lower than that between the islands (*F*_ST_ = 0.203) (Table 2). PCoA separated individuals between islands along Axis 2 but four individuals from Mayotte were clustered with individuals from Reunion (Figure 2C). In contrast, Axis 1, which accounted for a greater proportion of variance (26.7% vs. 13.2%), separated individuals according to depth: Eu and some UM individuals on one side and UM and LM individuals on the other (Figure 2C). However, this analysis did not clearly separate individuals by cryptic species. The additional PCoA showed that cryptic species appear to be segregated along Axis 1 in relation to depth (Figure S2). Cryptic species *β* was found in Eu and UM specimens from Reunion and Mayotte (the lone LM specimen from Mayotte was also a *β*). According to the PCoA, individuals were separated by genetic cryptic species based on a threshold along Axis 1 (settled at 0.14, Figure 2C, Figure S1). A total of 80 individuals of *M. phoenicea* were assigned to cryptic species *⍺* (86% of concordant morphological identification) and 82 as *β* (77%). Using this factor, the differentiation value between the two cryptic species was higher than with the morphological identification (*F*_ST_ = 0.180, *p*‐value < 0.001). Parts of the genetic variance estimated using AMOVA were first by island (29.7%, *p*‐value < 0.001), then by genetic cryptic species (28.6%, *p*‐value < 0.001), depth (14.4%, *p*‐value < 0.001) and finally by morphological cryptic species (12.8%, *p*‐value < 0.001).

The likely number of populations was *K* = 2 for simulations with islands as prior or without prior (Tables S1 and S2). However, the two clusters were not related to the island population: the first contained the Mayotte individuals from both euphotic and mesophotic depths, with some Reunion individuals from euphotic depths, and the second contained Reunion individuals from mesophotic depths and some euphotic individuals from Reunion (Figures 3C and 4B). In addition, several Reunion and Mayotte individuals had admixed profiles with admixture between the two clusters, suggesting a complex structure and hybridization in the *M. phoenicea* species complex (Figures 3C and 4B). With morphological cryptic species as prior, the likely numbers of populations estimated were *K* = 2 and *K* = 4 by Evanno's Δ*K*, and *K* = 4 by the log likelihood Ln Pr(*X*|*K*) (Figure S1, Table S2). The assignment for *K* = 2 with morphological cryptic species as prior (Figure 4C) was similar to that with islands as prior or without prior (Figures 3C and 4B), showing that clusters were not clearly assigned to cryptic species. All individuals of cryptic species *⍺* from the mesophotic depth of Reunion were assigned to one cluster, but individuals of the other cryptic species were also in the same cluster (Figure 4C). For *K* = 4, individuals were first separated by islands, with two clusters each for Mayotte and Reunion (Figure 4D). For the Mayotte clusters, admixture between individuals from mesophotic and euphotic depths was observed. Similarly, admixtures between cryptic species and sampling depths were observed for the Reunion clusters, with a tendency to segregate mesophotic individuals of cryptic species *⍺* and euphotic individuals of cryptic species *β* (Figure 4D). With genetic cryptic species as prior, both methods estimated the likely number of populations to be *K* = 2 (Tables S1 and S2). The two populations assigned in the structure analysis were assumed to correspond to the cryptic species *⍺* and *β* (Figure 4E). This population assignment was compared with the PCoA assignment of individuals to assess whether the Structure analysis was supported. As with morphological species as prior, individuals of cryptic species *⍺* from the mesophotic depths of Reunion were supported by PCoA and Structure assignments (Figure 4E). Meanwhile, the other clusters contained individuals with unsupported assignments, such as the four individuals from Mayotte who are assigned as *β* by PCoA and *⍺* by Structure (Figure 4E). Additionally, this assignment also had admixed profiles, which are ambiguous to compare with PCoA results, suggesting again the complex structure and hybridization in this species complex (Figure 4E).

### 3.4 Genetic Differentiation and Structure in the *Taxella* Species Complex

**FIGURE 5:**
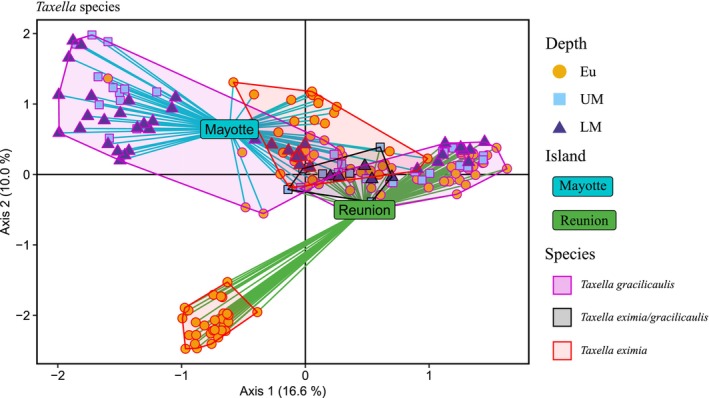
Principal coordinate analysis (PCoA) of *Taxella* species. Individuals are colored according to species: *Taxella gracilicaulis* in magenta, *Taxella eximia* in red, *Taxella eximia/gracilicaulis* in black, and by depth: Eu = euphotic in yellow, UM = upper mesophotic in light blue, and LM = lower mesophotic in dark blue. Individuals are connected to island centroids by colored lines, cyan for Mayotte, green for Reunion. Variance explained by the axis is given in brackets.

Globally, the two *Taxella* species were genetically separated (*F*_ST__*Te*‐*Tg* = 0.07, *p*‐value < 0.001) with 91 and 33 private alleles for *T. gracilicaulis* and *T. eximia* , respectively. The PCoA result showed that the three species groups (*T. gracilicaulis*, *T. eximia* , *T. eximia/gracilicaulis*) were closely related, except for the Reunion individuals of *T. eximia* (Figure 5). The *T. eximia/gracilicaulis* specimens and the Mayotte individuals of *T. eximia* were intermediate between the Mayotte and Reunion individuals of *T. gracilicaulis* (Figure 5), and the same result was obtained when the Reunion outgroup of *T. eximia* was removed (Figure S3). When considering only *T. gracilicaulis*, three distinct groups were observed: one for each island, constituted only by individuals from Reunion or Mayotte, and a third group mixing individuals from Reunion and Mayotte, mainly from euphotic depths (Eu), but also from upper (UM) and lower (LM) mesophotic depths (Figure S4).

Genetic differentiation values between islands were similar and significant for *T. gracilicaulis* and *T. eximia* (*F*_ST_ = 0.21, Table 2). The two *Taxella* species were also genetically distinct within islands, with *F*_ST__*Te*‐*Tg* = 0.18 (*p*‐value < 0.001) in Reunion and 0.15 (*p*‐value < 0.001) in Mayotte. For *T. gracilicaulis*, the genetic differentiation values between depths were all significant for both Mayotte and Reunion (Table 3). In Reunion, the *F*_ST_ between distant depths (*F*_ST__Eu‐LM = 0.07) was higher than those between adjacent depths (*F*_ST__Eu‐UM = 0.037 and *F*_ST__Eu‐LM = 0.039). However, these values remained lower than those in Mayotte, where a strong genetic differentiation was observed between the euphotic and mesophotic depths (*F*_ST__Eu‐UM = 0.165 and *F*_ST__Eu‐LM = 0.233) and between upper and lower mesophotic depths (*F*_ST__UM‐LM = 0.060) (Table 3).

**FIGURE 6:**
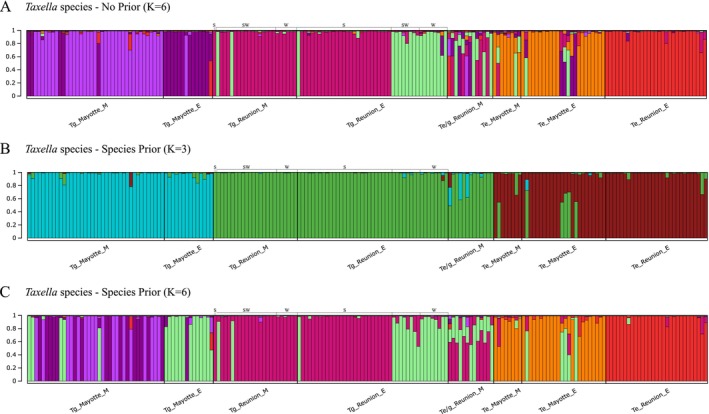
Assignment plots for *Taxella* species without prior and with Island Prior using the highest peaks of Δ*K* (Tables S1 and S2). (A) Structure assignment plots without prior for *K* = 6, (B–C) assignment plots with Island prior: (B) with *K* = 3 and (C) *K* = 6. Individuals are sorted by species, by island, and by depth. Individuals from Reunion are sorted by coast: S = south, SW = southwest, W = west.

Evanno's Δ*K* and log likelihood Ln Pr(*X*|*K*) methods estimated six populations without prior (*K* = 6). With species as prior, Evanno's Δ*K* estimated three populations (*K* = 3), but Ln Pr(*X*|*K*) method did not allow for a clear estimate (Figure S1, Tables S1 and S2). For *K* = 3, the assumed clusters corresponded to the populations of the species: (1) *T. gracilicaulis* individuals from Mayotte, (2) *T. gracilicaulis* individuals from Reunion with the *T. eximia/gracilicaulis* specimens, and (3) *T. eximia* individuals from Mayotte and Reunion (Figure 6B). For *K* = 6, the results were similar with and without species as prior (Figure 6A,C), but the assignment of individuals was clearer without prior (Figure 6A). The six assumed populations corresponded to the individuals of (1) mesophotic *T. gracilicaulis* from Mayotte, (2) euphotic *T. gracilicaulis* from Mayotte, (3) mesophotic and some euphotic *T. gracilicaulis* from Reunion, (4) euphotic *T. gracilicaulis* from the southwest (SW) and west (W) coasts of Reunion, (5) *T. eximia* from Mayotte, and (6) *T. eximia* from Reunion (Figure 6A). Moreover, admixtures between several populations were observed for the different species, in particular for the *T. eximia* population from Mayotte, which contained individuals associated with Reunion or Mayotte populations of *T. gracilicaulis* (Figure 6A). Although their morphological identifications were closer to *T. eximia* species, the *T. eximia/gracilicaulis* individuals were assigned to the *T. gracilicaulis* cluster for *K* = 3 (Figure 6B) and were not related to any *T. eximia* individuals from Mayotte or Reunion, either without or with species as prior (Figure 6A,C).

## 4 Discussion

This study estimated the genetic diversity and population structure of seven hydroid species between Mayotte and Reunion islands in the SWIO using microsatellite markers, across both euphotic and mesophotic depths. Our results revealed strong genetic differentiation between the two islands, along with contrasting levels of genetic diversity. Notably, cryptic diversity was suggested by the distinction between mesophotic and euphotic populations for some of the species (*L. phyteuma*, *M. phoenicea*, *T. eximia* /*gracilicaulis*, *Z. rufa* ), highlighting the potential influence of depth on population divergence in this regional context.

### 4.1 Contrasted Genetic Diversity Between Islands in the Southwestern Indian Ocean

Overall, allelic diversity and the number of private alleles were higher in Mayotte than in Reunion, despite fewer samples collected in Mayotte. The only exception was *M. phoenicea*, which showed lower allelic diversity in Mayotte, probably related to the presence of two cryptic species (Postaire et al. 2016). Previous studies of *L. brevirostris* and *M. phoenicea* showed similar results (Postaire et al. 2016; Postaire, Gélin, Bruggemann, and Magalon 2017; Postaire, Gélin, Bruggemann, Pratlong, and Magalon 2017), and additional genetic studies of hydroids and scleractinian corals showed higher genetic diversity in northern Madagascar (including the Comoros Archipelago) than in the Mascarenes (Boissin et al. 2018; van der Ven et al. 2021, 2022).

The contrasting genetic diversities can be attributed to multiple factors: life‐history strategies, demographic history, evolutionary process, etc. (Pauls et al. 2013; Alcala and Vuilleumier 2014; Romiguier et al. 2014; Ellegren and Galtier 2016; Manel et al. 2020). In our case, several hypotheses could explain the globally higher genetic diversity in Mayotte than in Reunion. First, anthropogenic pressures are known to affect the genetic diversity of natural populations (Ayre and Hughes 2004; DiBattista 2008). Reunion reefs have been severely affected by natural and anthropogenic pressures in recent decades with a global low coral cover of 20% (Bigot et al. 2000; Chabanet et al. 2002; Ahamada et al. 2008; Broudic et al. 2024), whereas Mayotte reefs, with a coral cover of 80%, may have better preserved genetic diversity (Wickel et al. 2015; Gudka et al. 2023). However, the lack of genetic diversity monitoring does not allow us to conclude on this hypothesis.

Another factor is oceanographic isolation. The Mascarene Islands, located at the southern boundary of the South Equatorial Current, are more isolated from regional currents compared with the northern Mozambique Channel (NMC), which receives influences from Madagascar, East Africa, and the northern Western Indian Ocean (Schott et al. 2009; Vogt‐Vincent and Johnson 2023). This connectivity contributes to the NMC's recognition as the heart of the Western Indian Ocean biodiversity hotspot, whereas the Mascarenes remain relatively isolated (Obura 2012; Hoareau et al. 2013; McClanahan et al. 2014; Fauvelot et al. 2020).

Finally, the historical evolution of the region may explain the observed genetic diversity patterns. The origin of the Western Indian Ocean hotspot dates back to the Eocene (~56 million years (Ma) ago) when it was connected to the highly diverse Tethys Ocean (Renema et al. 2008; Leprieur et al. 2016; Hou and Li 2018). Over time, the Tethys Ocean closed (~20 Ma) and later, the Indian and Pacific Oceans were separated by the formation of the Indo‐Australian Archipelago (~6 Ma), which is the most diversified region nowadays (Renema et al. 2008; Leprieur et al. 2016; Hou and Li 2018). Mayotte emerged ~9 Ma ago in the NMC (Nougier et al. 1986), while Reunion emerged ~3 Ma ago (McDougall 1971; Camoin et al. 2004). More recently, the NMC is thought to have acted as an evolutionary hotspot in the fragmented Western Indian Ocean. During the last glacial period in the Miocene (18,000–17,000 years ago), sea level dropped down to 110–115 m below the present level leading to habitat fragmentation and loss of connectivity between islands (Camoin et al. 2004). This historical evolution is supported by occurrences of speciation events (Hoareau et al. 2013; Fauvelot et al. 2020; Li et al. 2021) and may have allowed the emergence and maintenance of high genetic diversity in Mayotte, but not in Reunion.

### 4.2 Population Structure and Limited Horizontal Connectivity

In this study, numerous private alleles and significant genetic differentiation between islands were estimated for the seven species, supported by PCoAs and Structure assignments. This finding suggests a lack of connectivity between these two islands in the SWIO. Our results for *M. phoenicea* and *L. brevirostris* confirm those obtained in previous studies where weak connectivity was shown between populations from the Mascarene Islands and the northwestern Mozambique Channel (Postaire et al. 2016; Postaire, Gélin, Bruggemann, and Magalon 2017; Postaire, Gélin, Bruggemann, Pratlong, and Magalon 2017). In addition, mitochondrial DNA analysis (16S gene) of some species (*L. phyteuma*, *T. gracilicaulis*, *Z. rufa* ) confirmed the absence of shared haplotypes between islands (Boissin et al. 2018). These results are consistent with previous population genetic studies which estimated strong genetic differentiation between remote regions of the SWIO for coral species (van der Ven et al. 2021, 2022), and between the Mascarene and Comoros islands for hydroids and scleractinian corals (Postaire et al. 2016; Postaire, Gélin, Bruggemann, and Magalon 2017; Postaire, Gélin, Bruggemann, Pratlong, and Magalon 2017; Boissin et al. 2018; Oury et al. 2024). This lack of connectivity is largely attributed to the complex oceanographic circulations in the region (Schott et al. 2009; Vogt‐Vincent and Johnson 2023). As mentioned above, the south equatorial current is a major current of the SWIO that isolates the Mascarene archipelago from the NMC. The south equatorial current and the formation of gyres in the NMC create a barrier to gene flow, preventing the spread of propagules from the western (NMC) to the eastern (Mascarenes) part of the SWIO. Modeling studies of connectivity in the SWIO are consistent, estimating limited connectivity in the short term, but not over multi‐generational or evolutionary timescales (Crochelet et al. 2016; Gamoyo et al. 2019; Vogt‐Vincent et al. 2024).

On a broader Indo‐Pacific scale, genetic structure was observed between *L. phyteuma* populations in the SWIO (Reunion and Mayotte) and in the Pacific Ocean (Moorea, French Polynesia), supported by *F*_ST_, PCoA, and Structure analyses. This result is consistent with the lack of connectivity between the Indian and Pacific Oceans observed for the hydroids *L. brevirostris* and *M. phoenicea* (Postaire et al. 2016; Postaire, Gélin, Bruggemann, and Magalon 2017; Postaire, Gélin, Bruggemann, Pratlong, and Magalon 2017), as well as other benthic taxa (Hoareau et al. 2012; Oury et al. 2021). The lack of connectivity between the Pacific Ocean and the SWIO may be explained by two strong barriers to gene flow. First, the Indo‐Australian Archipelago, which acts as a barrier between the Pacific and Indian Oceans for numerous organisms (Carpenter and Springer 2005; Gaither and Rocha 2013; Bowen et al. 2016). The second is the thousands of kilometers of open ocean between the eastern and western parts of the Indian Ocean (Schott et al. 2009; Vogler et al. 2012; Otwoma and Kochzius 2016; Cacciapaglia et al. 2021).

Distinguishing between intraspecific genetic structure and true cryptic speciation remains difficult, as the line separating the two is often blurred. Studies on hydroids have shown that cryptic species are often found in widespread species (Moura et al. 2008). For example, cryptic species have been reported for *Pteroclava krempfi* in the Caribbean and Maldives (Maggioni et al. 2016; Montano et al. 2017) and for the *Plumularia setacea* species complex (Schuchert 2014). Cryptic species were also identified among SWIO hydroids, using both mitochondrial (16S gene), conserved nuclear (calmodulin gene), or microsatellite markers (Postaire et al. 2016; Postaire, Gélin, Bruggemann, and Magalon 2017; Postaire, Gélin, Bruggemann, Pratlong, and Magalon 2017; Boissin et al. 2018). These studies support the “one island, one species” hypothesis, suggesting fine‐scale endemism, where each island or island group hosts a distinct genetic lineage (Postaire et al. 2016; Postaire, Gélin, Bruggemann, and Magalon 2017; Postaire, Gélin, Bruggemann, Pratlong, and Magalon 2017; Boissin et al. 2018).

### 4.3 Population Structure and Connectivity Between Euphotic and Mesophotic Depths

Vertical population structure in the SWIO was suggested by PCoAs and Structure assignments for two species: *L. phyteuma* and *Z. rufa* . For *L. phyteuma*, strong vertical differentiation between mesophotic and euphotic depths was supported by pairwise *F*_ST_ values in Mayotte, but not in Reunion. However, *L. phyteuma* is rarely found at mesophotic depths in Reunion, with only 10 samples collected after 50 mesophotic dives, compared with 14 samples obtained after only seven mesophotic dives in Mayotte. This suggests that mesophotic habitats in Reunion might not be suitable for this species contrary to mesophotic habitats in Mayotte. Reunion island is young (McDougall 1971; Camoin et al. 2004), and its mesophotic coral ecosystems are close to the coast, which are highly anthropized (Ahamada et al. 2008; Broudic et al. 2024). In contrast, Mayotte Island is older (Nougier et al. 1986), and its mesophotic coral ecosystems are farther from the coast and potentially less affected by disturbances (Figure 1). Due to the different latitudes of the two islands, water temperature could also explain our result, but there is no temperature monitoring in the mesophotic zone to support this theory. For *Z. rufa* , the low number of euphotic samples from Mayotte prevented us from estimating F_ST_ values between depths. On Reunion, *Z. rufa* shows lower genetic differentiation values between adjacent depths (euphotic vs. upper mesophotic, upper vs. lower mesophotic) than between distant depths (euphotic vs. lower mesophotic). This vertical “step‐by‐step” pattern of connectivity is similar to the stepping stone pattern already described on the horizontal dimension (Kimura and Weiss 1964). The second prerequisite of vertical connectivity from the DRRH implicitly assumes that connectivity occurs from mesophotic to euphotic depths by bottom‐up gene flow (Bongaerts et al. 2010, 2017; Sturm et al. 2022, 2023). Then, a vertical stepping stone pattern is not incompatible with this prerequisite as long as there is bottom‐up gene flow. For *Z. rufa* on Reunion, analyses show bottom‐up gene flow from lower mesophotic to euphotic depths, which supports the DRRH prerequisite of vertical connectivity (Ory, Gravier‐Bonnet, et al. 2025). It will be interesting to go further and perform the same analyses on additional samples from Mayotte, especially in light of the results of the present study. Our results revealed vertical population structures for *L. phyteuma* and *Z. rufa* in the SWIO, highlighting the importance of including mesophotic samples in population genetic studies to better understand large‐scale connectivity.

Various patterns of vertical connectivity between taxa (Bongaerts et al. 2017) and regions (Brazeau et al. 2013; Serrano et al. 2014; Studivan and Voss 2018; Sturm et al. 2023) have been observed for scleractinian corals in the Caribbean Sea and the Great Barrier Reef. Similar results have been observed for hydroids in Reunion, with different patterns of connectivity between species and between different areas of the island (Ory, Gravier‐Bonnet, et al. 2025). In the context of euphotic reef degradation and the potential for mesophotic reefs to reseed shallower reefs (DRRH), further investigation of mesophotic connectivity across species and regions is essential.

### 4.4 Hidden Diversity and Structure in the *Taxella eximia*/gracilicaulis Species Complex

Complex patterns of genetic differentiation were observed for the *T. eximia* /*gracilicaulis* species complex, suggesting vertical genetic structure and differentiation between islands. Results revealed different levels of population structure among species, islands, and depths. First, for *T. gracilicaulis*, individuals were separated into three groups: two groups consisting of individuals from Reunion or Mayotte and a third group consisting of individuals from the two islands. In analyses involving all *Taxella* species, this third group was not clearly identified, as it was mixed with some *T. eximia* and hybrid individuals. In Mayotte, individuals were strongly separated between euphotic and mesophotic depths, whereas in Reunion, no depth‐related separation was noted. However, the euphotic individuals from the western and southwestern regions of Reunion were separated from the other *T. gracilicaulis*, suggesting the presence of local cryptic species. Genetic differentiation values were similar to those for *Z. rufa* in Reunion, supporting the vertical connectivity prerequisite of the DRRH (Ory, Gravier‐Bonnet, et al. 2025). Thus, *Taxella gracilicaulis* is a good candidate species to test this prerequisite of the DRRH in Reunion, but additional samples are required to perform gene flow analysis. Conversely, *T. eximia* displayed population structure between islands but not across depths in Mayotte, also making it a candidate species for the DRRH. In Reunion, euphotic samples of *T. eximia* are strongly separated from the other clusters, with no mesophotic samples obtained, suggesting its absence at such depths for an unknown reason. Future mesophotic sampling should prioritize *Taxella* species to continue testing the DRRH.

A previous systematic study of the *Gymnangium* and *Taxella* rehabilitated the *Taxella* genus but also revealed that the distinction between *T. gracilicaulis* and *T. eximia* species is not supported by single gene DNA barcoding analysis, despite morphological differences (Ronowicz et al. 2017). Genetic differentiation and cryptic species between regions of the SWIO have already been studied in this species complex (Boissin et al. 2018). However, both studies used only one mitochondrial marker (16S gene), which did not allow the separation of the two species. Here, microsatellite markers developed specifically for *T. gracilicaulis* and used by cross‐amplification on *T. eximia* (Ory, Mouronvalle, et al. 2025) provided better resolution of this species complex, successfully distinguishing the two species and misidentified individuals. Our results are encouraging because the microsatellite marker set successfully separated the species, including misidentified individuals, such as the *T. eximia/gracilicaulis* individuals that clustered with *T. gracilicaulis* although morphologically related to *T. eximia* . Furthermore, the results for these *T. eximia/gracilicaulis* individuals, considering that the two species and these intermediate forms were found in sympatry, suggest possible genetic hybridization between *T. gracilicaulis* and *T. eximia* . In addition, the results globally highlight a true species complex consisting of multiple, potentially hybridizing genetic groups and an underestimation of the diversity in this genus. These nuclear markers now allow further identification of potential cryptic *Taxella* species. Continued investigation using integrative taxonomy will be essential to accurately characterize species diversity within this complex and to elucidate the evolutionary forces at work.

Several systematic revisions of hydroid families have already been carried out, highlighting the underestimation of species diversity worldwide (Schuchert 2004; Cairns 2005; Moura et al. 2008, 2011a, 2011b, 2012, 2018; Maronna et al. 2016; Ronowicz et al. 2017; Miglietta et al. 2019; Galea et al. 2020; Galea and Maggioni 2024). Upon closer inspection, they also highlight paraphyly and polyphyly in several genera. Furthermore, studies at mesophotic and deep depths are followed by the discovery of new genera/species, adding further complexity (Gravier‐Bonnet et al. 2022; Gu et al. 2022; Maggioni et al. 2022). Integrative studies using powerful molecular markers, such as the microsatellites used here or single nucleotide polymorphisms (SNPs) in combination with morphological studies may be the key to resolving systematic incoherences in hydroids.

### 4.5 Mesophotic Samples Provide New Insights Into *M. phoenicea* Species Complex

For another known species complex, *M. phoenicea* (Postaire et al. 2016; Postaire, Gélin, Bruggemann, Pratlong, and Magalon 2017; Boissin et al. 2018), the addition of mesophotic samples from Reunion and Mayotte brings new insights to its understanding. The analyses did not clearly distinguish individuals by island, depth, or cryptic species, suggesting greater complexity than previously recognized. Previous studies found cryptic species *⍺* on different SWIO islands, while species *β* was restricted to Reunion, where the species are sympatric, sometimes at the same sampling sites (Postaire et al. 2016; Postaire, Gélin, Bruggemann, Pratlong, and Magalon 2017). Here, the cryptic species *β* was found on both Mayotte and Reunion, whereas the cryptic species *⍺* was found only on Reunion. In previous studies of *M. phoenicea* in the SWIO, no specimens of the cryptic species *β* were sampled in Mayotte (Postaire et al. 2016; Postaire, Gélin, Bruggemann, Pratlong, and Magalon 2017). However, the color or shape of the specimens is variable, leading to possible misidentifications, as supported by the different analyses (AMOVA, *F*_ST_, Structure) performed between morphological and genetic cryptic species. Here, our genetic results place the specimens identified as *β* from Mayotte closer to the cryptic species *β* from Reunion, suggesting that both species may be present on Mayotte. Our results also provide new insights for *M. phoenicea* on Reunion, with the cryptic species *⍺* found mainly at mesophotic depths and the cryptic species *β* at euphotic depths, but overlapping at certain depths (Ory, Gravier‐Bonnet, et al. 2025). These results suggest possible gene flow between the two cryptic species. The presumed clusters of individuals based on cryptic species (identified by morphology), islands, and sampling depths did not coincide with the estimated genetic groups (with the exception of the cryptic species *⍺* from mesophotic depths of Reunion).

Some aspects of the *Taxella* and *M. phoenicea* species complex results look like gray zones of speciation, suggesting that some cryptic species may currently be undergoing speciation (Roux et al. 2016; Dufresnes et al. 2023). The increase in mesophotic studies made possible by the democratization of exploration techniques (CCRs and remotely operated underwater vehicles) is leading, as here, to the discovery of new species (Muir et al. 2018; Pyle and Copus 2019; Tea et al. 2019; Gravier‐Bonnet et al. 2022; Anker et al. 2023) and cryptic diversity at these depths (Prata et al. 2022; Gijsbers et al. 2023; Eckert et al. 2024). Continued research efforts at mesophotic depths will lead to the discovery of new species and improve our understanding of evolutionary mechanisms at these depths.

## 5 Conclusion

This population genetic study based on microsatellite markers uncovers cryptic diversity between islands, lending strong support to the “one island, one species” hypothesis for hydroids. Our findings also suggest that cryptic diversity extends into mesophotic depths, highlighting the importance of considering multiple sites and depths in population genetic studies. Microsatellite markers have proven to be very effective for examining species complexes, for which the contribution of mesophotic samples brings new light, such as in the genus *Taxella*. Further integrative studies combining genetics, morphology, and ecology are needed to clarify hydroid systematics. The estimation of vertical connectivity suggests several putative species (*Lytocarpia brevirostris*, *Sertularella diaphana* , *Taxella eximia*, *Taxella gracilicaulis*, and *Zygophylax rufa*) as promising models to test the vertical connectivity prerequisite of the DRRH in the SWIO. Future research should aim to expand our understanding of mesophotic connectivity at broader spatial scales, while accounting for regional specificities in order to support the development of more effective and context‐sensitive conservation strategies.

## Author Contributions

**David Ory:** data curation (equal), formal analysis (lead), investigation (lead), methodology (lead), writing – original draft (lead). **Nicole Gravier‐Bonnet:** data curation (equal), formal analysis (supporting), investigation (equal), methodology (equal), validation (equal), writing – review and editing (equal). **Pascale Chabanet:** project administration (equal), resources (equal), supervision (supporting), writing – review and editing (equal). **Chloé A. F. Bourmaud:** conceptualization (equal), investigation (equal), methodology (equal), project administration (equal), supervision (equal), validation (equal), writing – review and editing (equal). **Emilie Boissin:** conceptualization (equal), data curation (equal), formal analysis (supporting), funding acquisition (lead), investigation (equal), methodology (equal), resources (equal), supervision (equal), validation (equal), writing – original draft (supporting), writing – review and editing (equal).

## Funding

This study was supported by the Inventaire National du Patrimoine Naturel (INPN), Laboratoire d'Excellence Corail, Agence Française de Développement, Office Français de la Biodiversité, European Regional Development Fund.

## Conflicts of Interest

The authors declare no conflicts of interest.

## Supporting information


**Appendix S1:** ece372665‐sup‐0001‐AppendixS1.zip.

**Figure S1:** Log likelihood plots for structure analyses of the seven species.

**Figure S2:** Principal coordinate analysis (PCoA) of *Macrorhynchia phoenicea* (*⍺* and *ꞵ*) from Figure 2C with the distinction of cryptic species of individuals.

**Figure S3:** Principal coordinate analysis (PCoA) of *Taxella* species excluding the outgroup of *Taxella eximia* from Reunion.

**Figure S4:** Principal coordinate analysis (PCoA) of *Taxella gracilicaulis*.

**Table S1:** Details of Evanno's Δ*K* and log likelihood results for structure analyses without prior.

**Table S2:** Details of Evanno's Δ*K* and log likelihood results for structure analyses with prior.

## Data Availability

Sample information, multilocus genotypes, and GPS coordinates are provided in Data S1. Script of R packages and functions used for analyses are given in Data S2. Files are available at: https://zenodo.org/records/17360631?token=eyJhbGciOiJIUzUxMiJ9.eyJpZCI6IjAyNzc5YWFmLTVmYTgtNGRjNy1iOWJkLTFmNWI0ODNkYzU1ZSIsImRhdGEiOnt9LCJyYW5kb20iOiJlMTJlNjJiODYzZjBhZGNkMzYxOWZmMzY0NmM5YjNlOCJ9.‐neTOYl7Qbjhpkx7SAST9GMGr3uAfwTvRLK1dEvoxHXH4ZgrM70L3jnD9UO_8Lsqo_Ghitd21HR8tf0BWkrx5g.
