# Supplementary material for: Cryptic Diversity and Genetic Differentiation of Mesophotic Hydroids in the Southwestern Indian Ocean
Source: Ecol Evol. 2025 Dec 17;15(12):e72665. doi: 10.1002/ece3.72665 (PMC12710443; doi:10.1002/ece3.72665)
Supplement: Supplementary file 2 — Figure S1: Log likelihood plots for structure analyses of the seven species. [file ECE3-15-e72665-s006.pdf]

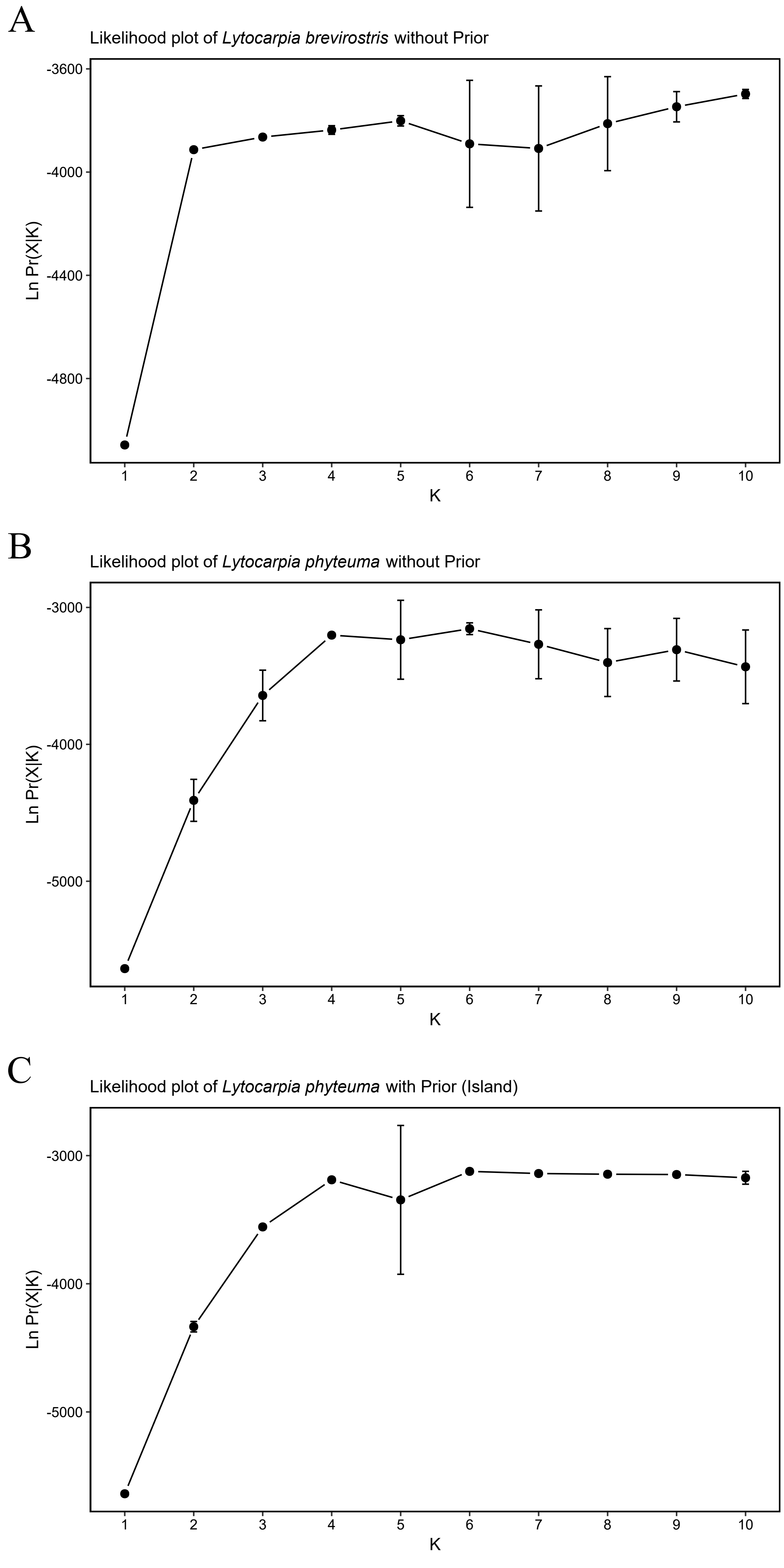
A
Likelihood plot of *Lytocarpia brevirostris* without Prior
-3600
-4000
-4400
-4800
Ln Pr(X|K)
1
2
3
4
5
6
7
8
9
10
K
B
Likelihood plot of *Lytocarpia phyteuma* without Prior
-3000
-4000
-5000
Ln Pr(X|K)
K
C
Likelihood plot of *Lytocarpia phyteuma* with Prior (Island)
-3000
-4000
-5000
Ln Pr(X|K)
K

D

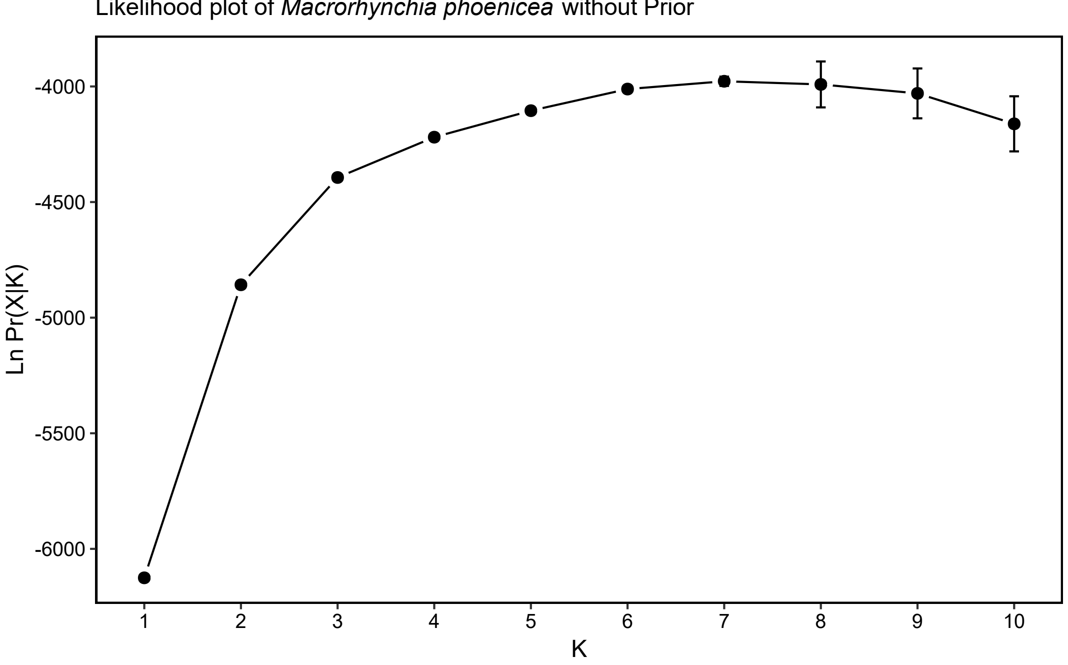
Likelihood plot of Macrorhynchia phoenicea without Prior
Ln Pr(X|K)
-4000
-4500
-5000
-5500
-6000
1 2 3 4 5 6 7 8 9 10
K

E

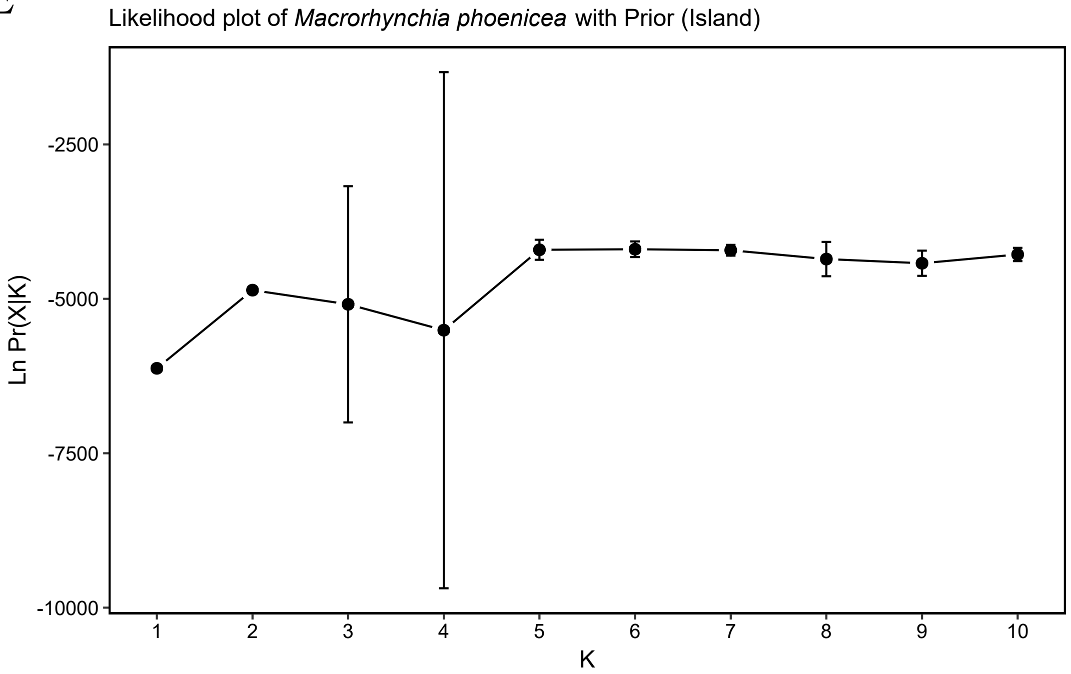
Likelihood plot of Macrorhynchia phoenicea with Prior (Island)
Ln Pr(X|K)
-2500
-5000
-7500
-10000
1 2 3 4 5 6 7 8 9 10
K

F

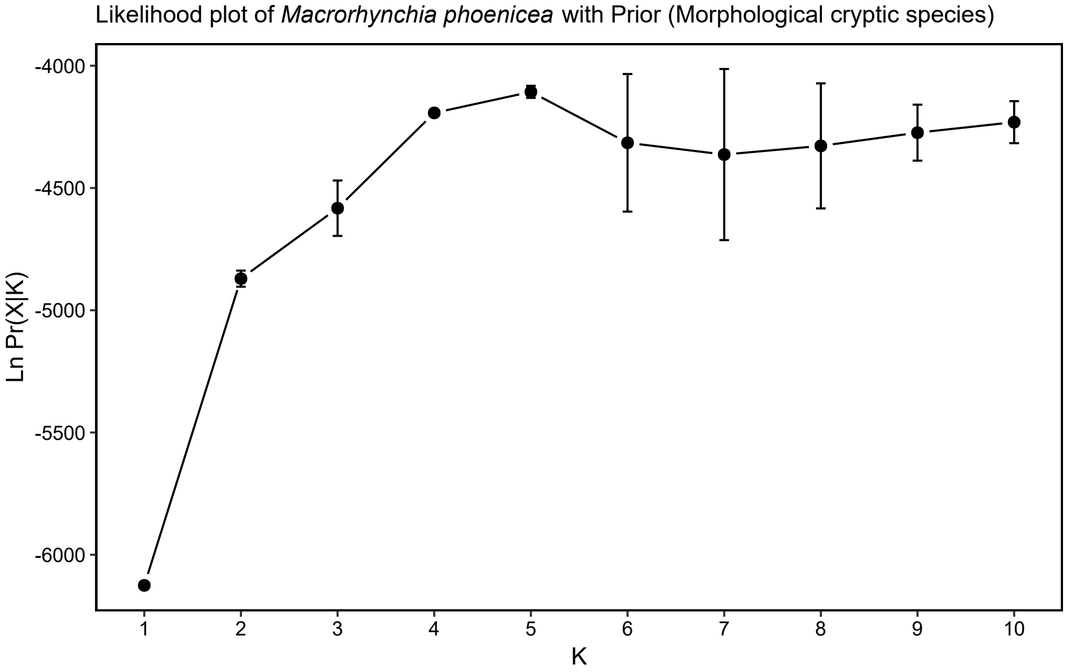
Likelihood plot of Macrorhynchia phoenicea with Prior (Morphological cryptic species)
Ln Pr(X|K)
-4000
-4500
-5000
-5500
-6000
1 2 3 4 5 6 7 8 9 10
K

G

Likelihood plot of *Macrorhynchia phoenicea* with Prior (Genetic cryptic species)

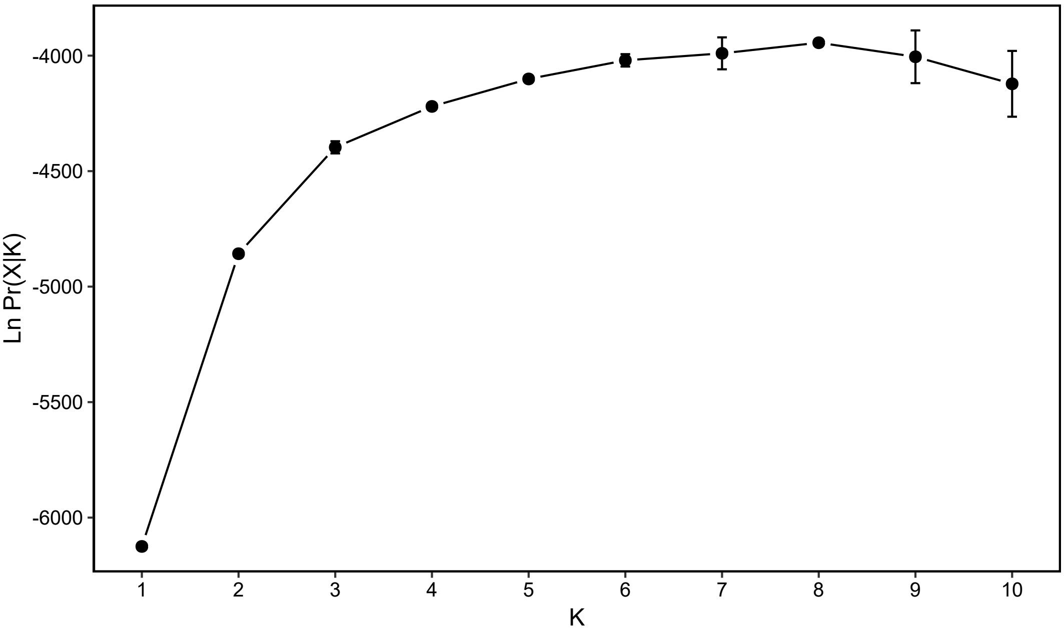


H

Likelihood plot of *Sertularella diaphana* without Prior

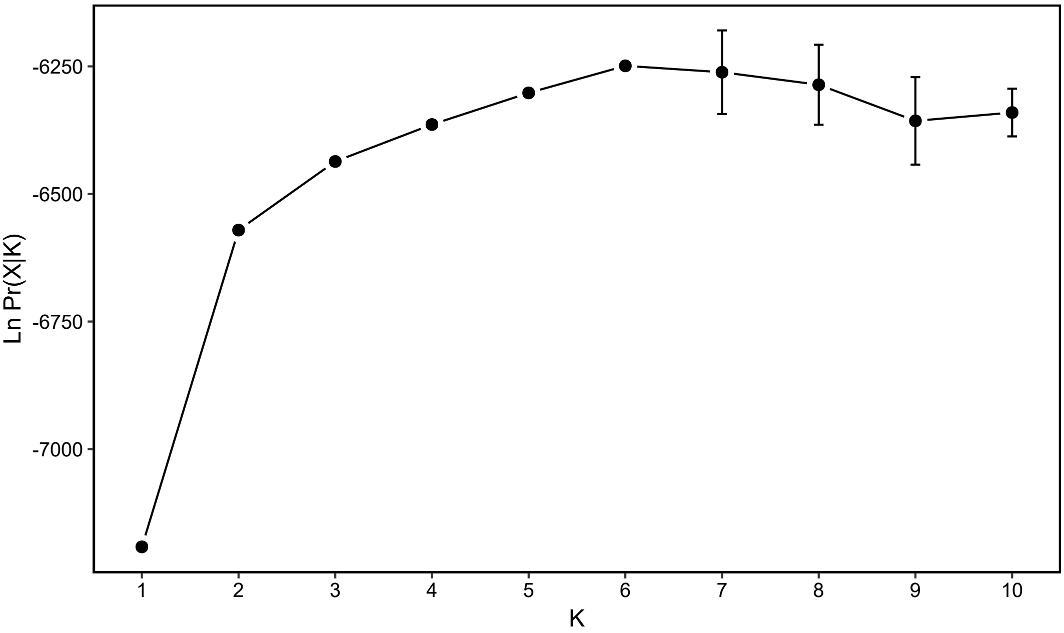


I

Likelihood plot of *Taxella* species without Prior

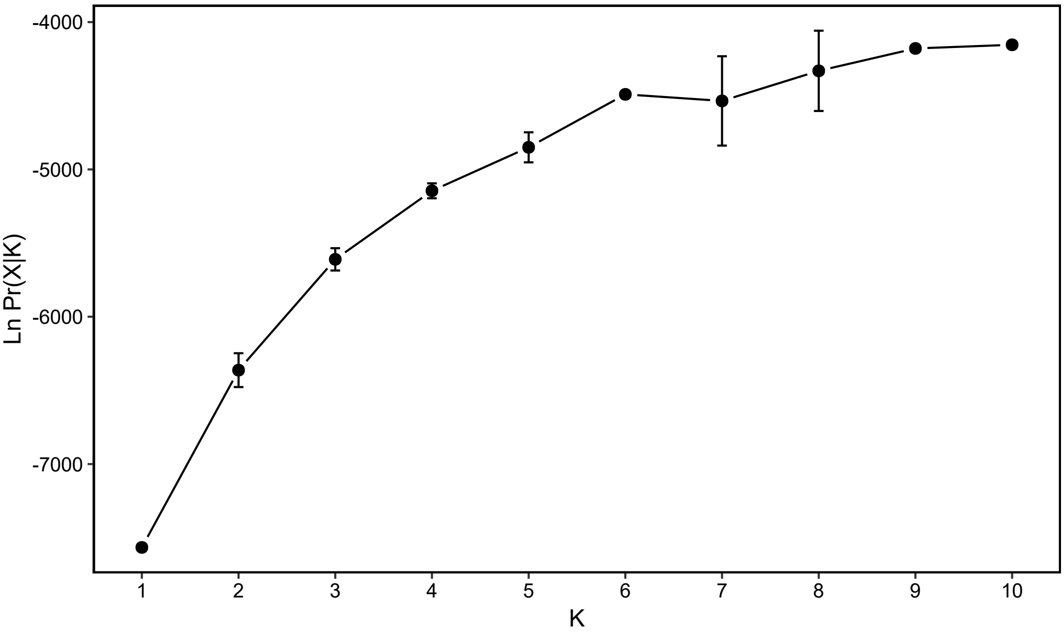

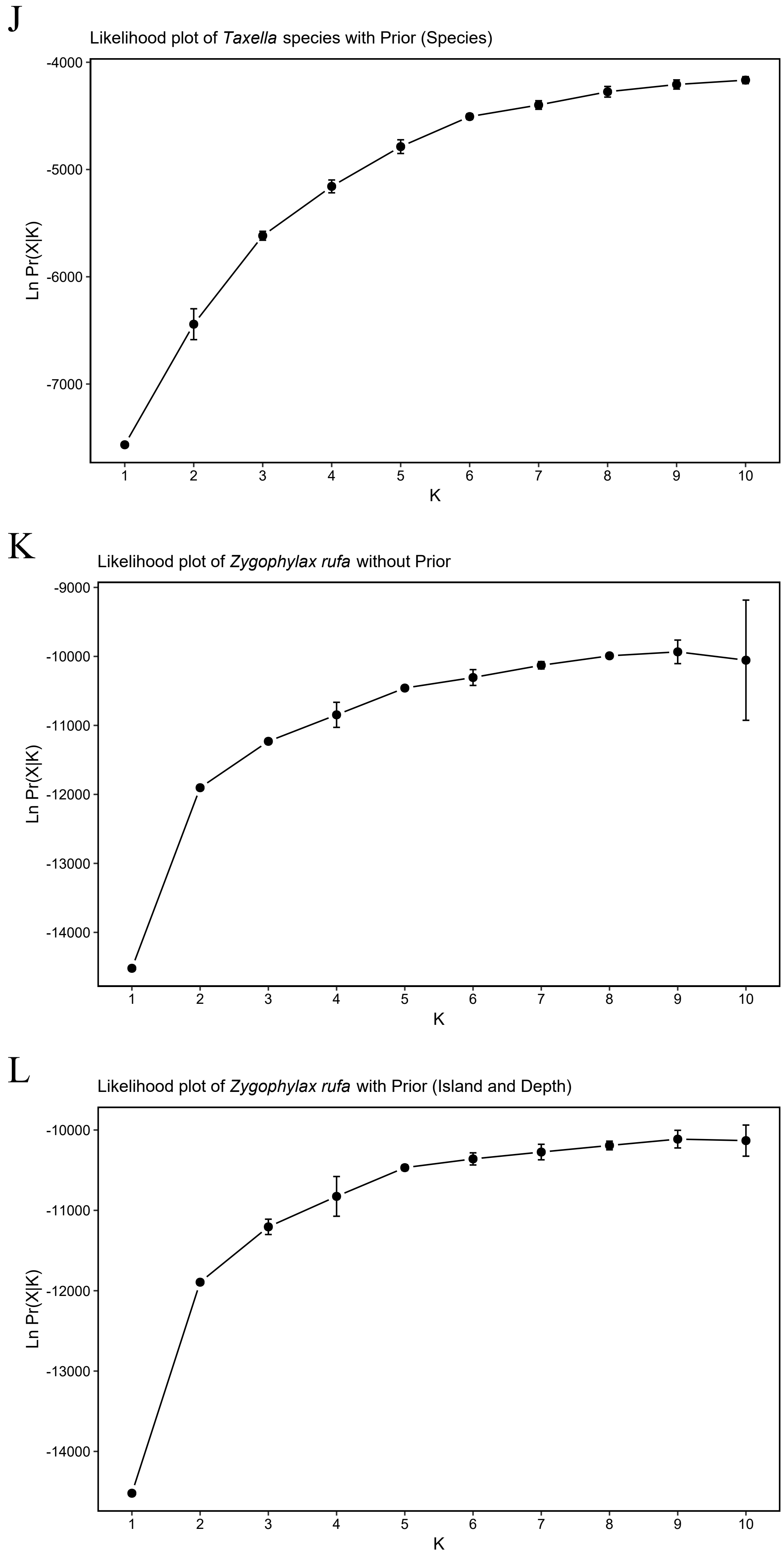
J
Likelihood plot of *Taxella* species with Prior (Species)
-4000
-5000
-6000
-7000
Ln Pr(X|K)
1
2
3
4
5
6
7
8
9
10
K
K
Likelihood plot of *Zygophylax rufa* without Prior
-9000
-10000
-11000
-12000
-13000
-14000
Ln Pr(X|K)
1
2
3
4
5
6
7
8
9
10
K
L
Likelihood plot of *Zygophylax rufa* with Prior (Island and Depth)
-10000
-11000
-12000
-13000
-14000
Ln Pr(X|K)
1
2
3
4
5
6
7
8
9
10
K
