# Supplementary figures and images for: Cryptic Diversity and Genetic Differentiation of Mesophotic Hydroids in the Southwestern Indian Ocean
Source: Ecol Evol. 2025 Dec 17;15(12):e72665. doi: 10.1002/ece3.72665 (PMC12710443; doi:10.1002/ece3.72665)

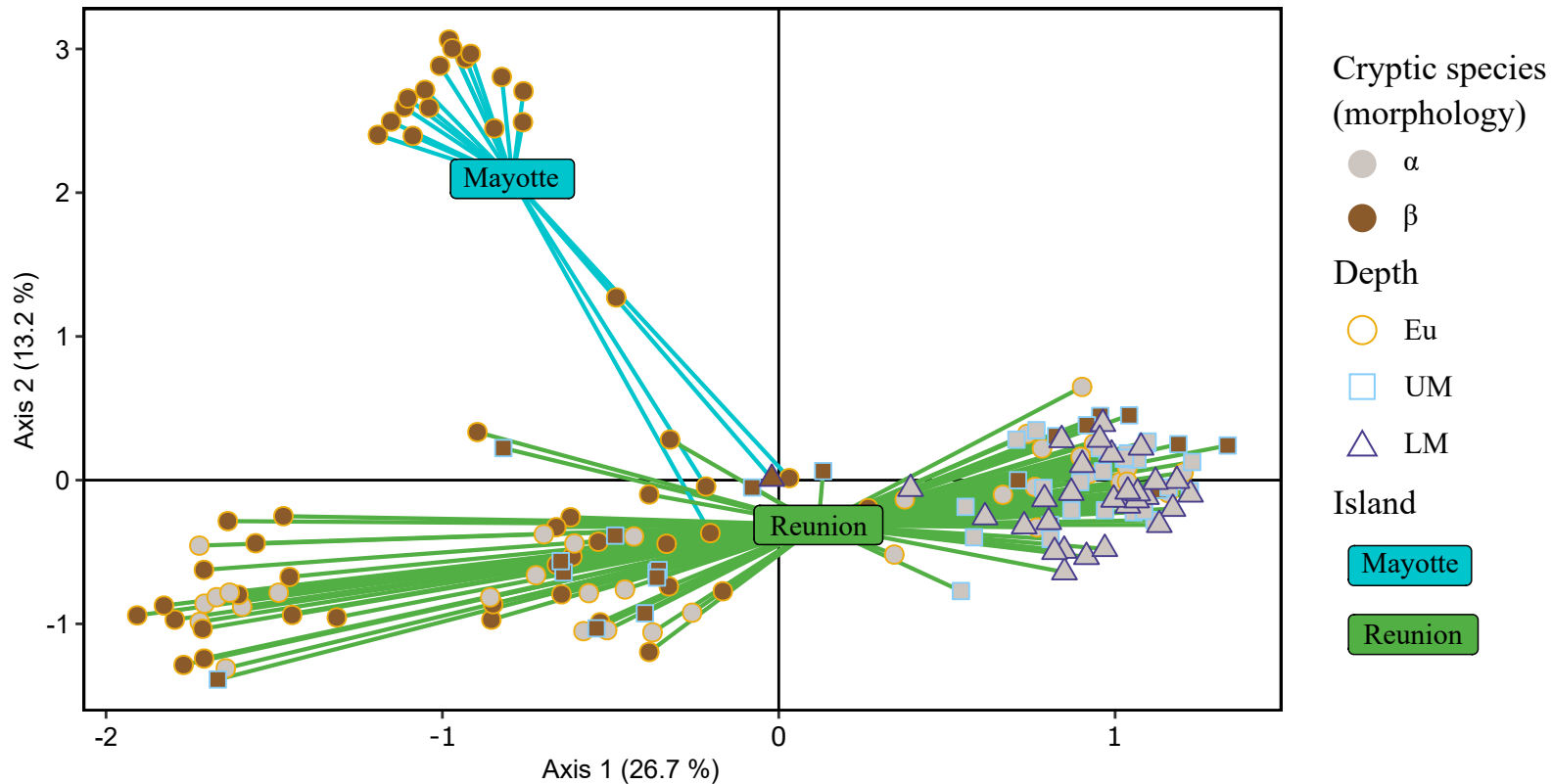

Supplement: Supplementary file 3 — Figure S2: Principal coordinate analysis (PCoA) of Macrorhynchia phoenicea (⍺ and ꞵ) from Figure 2C with the distinction of cryptic species of individuals. [file ECE3-15-e72665-s005.pdf]

*Taxella* species

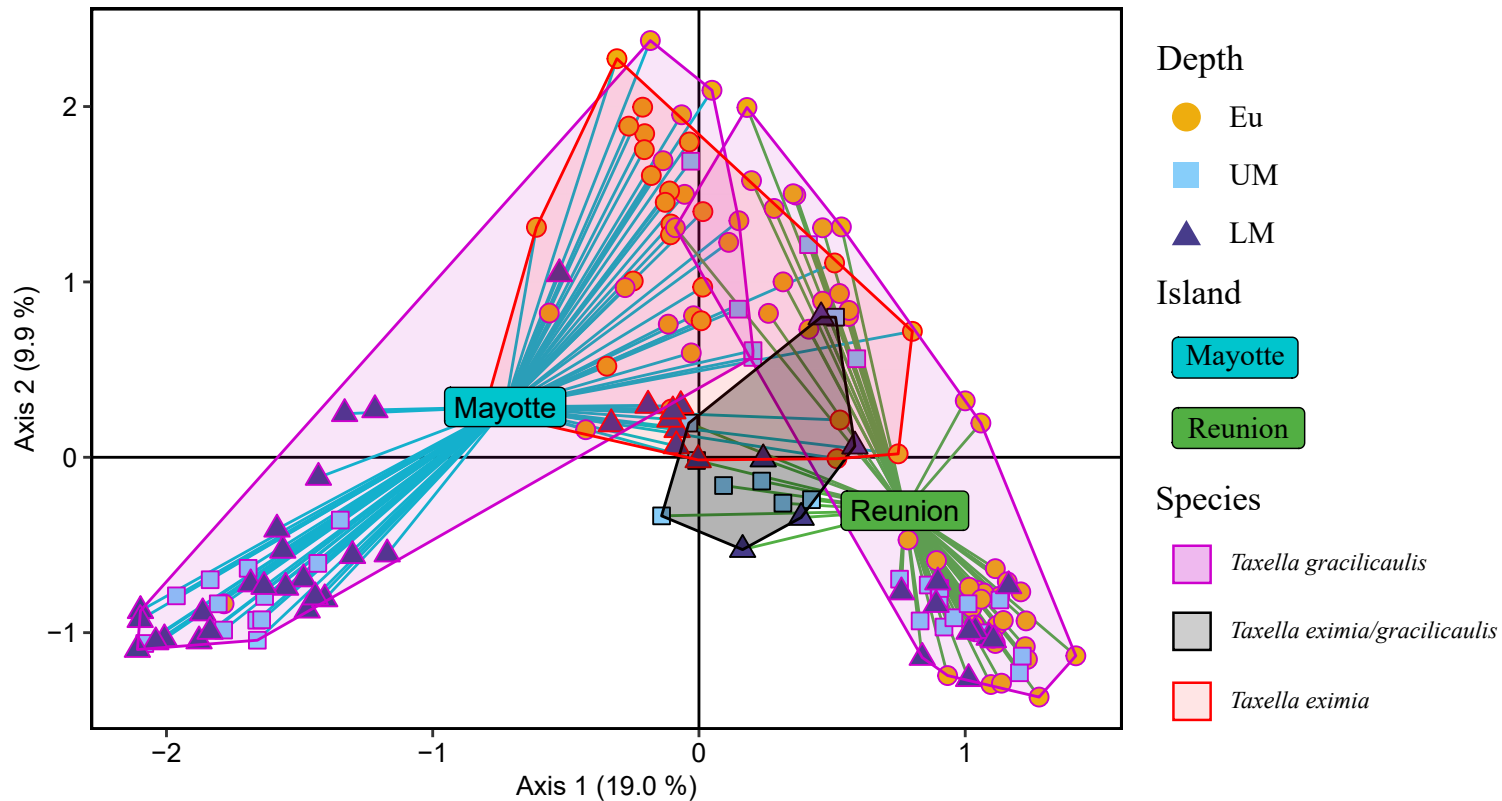

Supplement: Supplementary file 4 — Figure S3: Principal coordinate analysis (PCoA) of Taxella species excluding the outgroup of Taxella eximia from Reunion. [file ECE3-15-e72665-s004.pdf]

*Taxella gracilicaulis*

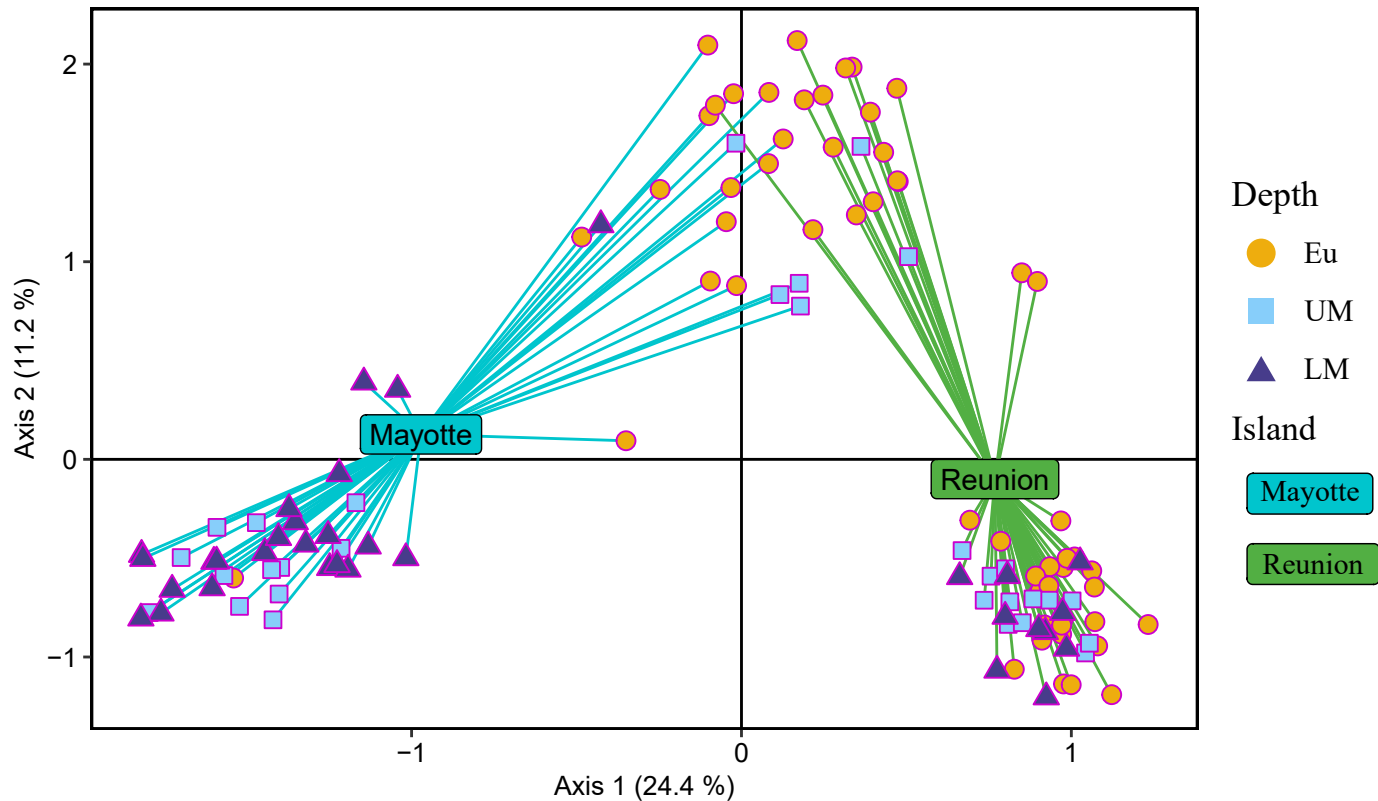

Supplement: Supplementary file 5 — Figure S4: Principal coordinate analysis (PCoA) of Taxella gracilicaulis. [file ECE3-15-e72665-s002.pdf]
